# Efficiency, market concentration and bank performance during the COVID-19 outbreak: Evidence from the MENA region

**DOI:** 10.1371/journal.pone.0285403

**Published:** 2023-05-10

**Authors:** Miroslav Mateev, Muhammad Usman Tariq, Ahmad Sahyouni

**Affiliations:** 1 Abu Dhabi School of Management, Abu Dhabi, UAE; 2 Higher Institute for Administrative Development, Damascus University, Damascus, Syria; Bucharest University of Economic Studies: Academia de Studii Economice din Bucuresti, ROMANIA

## Abstract

This study aims to contribute to the existing literature that explores the impact of market concentration on bank efficiency in emerging economies. Using a sample of 225 banks in 18 countries in the Middle East and North Africa (MENA) region over the period 2006–2020, we empirically investigate the significance of this relationship. Since the evidence of concentration effect on efficiency during the COVID-19 outbreak is ambiguous, we test the hypothesis that the efficiency is positively affected by the level of banking market concentration in the MENA region. We adopt fixed effect model specifications and test the robustness of our results with the two-step Generalized Method of Moments (GMM) estimation technique. Our analysis finds a strong positive association between market concentration and bank efficiency. The analysis of different types of banking systems that co-existing in the MENA region (Islamic and conventional) indicates the market concentration effect is more pronounced when the banking institution is Islamic and during the COVID-19 outbreak. Moreover, the better economic performance of Islamic banks during the initial stage of pandemic further increases their efficiency. Our analysis indicated that the impact of market competitive conditions on bank efficiency varies significantly across banks with different ownership structures and is more pronounced for government-owned banks. The results are robust using different model specifications and alternative estimation techniques.

## Introduction

In this paper, we investigate the effect of market concentration on bank efficiency using a sample of 225 banks in the countries in the Middle East and North Africa (MENA) region. According to Kozak and Wierzbowska [1], the importance of the relationship between banking market concentration and bank efficiency lies in the fact that “bank efficiency affects the ability to extend loans and ensure the financial stability of the entire banking sector” (p. 1). This issue is significant for emerging economies, including those in the MENA region, where financial markets are still underdeveloped, and bank credit is the primary source of financing economic growth. Therefore, investigating the relationship between market concentration and bank efficiency in the context of the current global (COVID-19) crisis is an important issue for both researchers and policymakers.

Empirical literature investigating the effect of banking market structure on efficiency provides conflicting evidence. While some studies report a positive impact on bank efficiency reflected in more favorable prices and higher income [2, 3] and/or significant reduction in operating costs, others find that market concentration is the main reason for a decline in bank efficiency and earnings [4, 5]. This ambiguous relationship between concentration and bank efficiency remains a great puzzle. It requires additional analysis of other factors that may impact efficiency, for instance, a “strict control over interest rates on loans and deposits rather than the use of a monopoly profits” [6, p. 50]. We add to the existing empirical literature that explores the relationship between banking market structure and efficiency by analyzing this relationship for banks with different ownership structures (government-owned and foreign-owned) and different banking systems (Islamic vs. conventional) that exist in the MENA region. Specifically, we address the following three questions: Is the efficiency level positively or negatively affected by the banking market concentration? What is the effect of the COVID-19 pandemic on bank efficiency? How different is the concentration effect on the efficiency of Islamic and conventional banks?

Recently, a considerable number of studies have focused on assessing the impact of COVID-19 on the banking systems of different countries and/or regions [7–9]. The empirical research on bank efficiency and performance in the MENA region has been growing as well. Three main reasons make this region an interesting laboratory for research. First, we have witnessed a significant impact of the COVID-19 crisis on the MENA region’s countries, leading to a “significant fall in oil production, a strong adverse effect on hospitality and tourism, and a decline in the GDP per capita income” [10, p. 53]. Second, the findings of previous studies on the developed countries of North America and Western Europe [11, 12] arguably limit their generalization to MENA countries because there are substantial differences in terms of financial, political, regulative, and economic features, among others, between developed and developing countries, and even within the MENA region [13]. Third, the link between Islamic finance and long-terms economic growth and stability in the MENA region became an important question for both policy makers and researchers. Another notable aspect is the economic impact and role of Islamic finance in the MENA region, which faces a spectacular growth in recent years [14]. Bitar et al. [15] stated that the rapid development in this sector is triggered by at least two factors: “(i) oil revenues from Gulf countries, and (ii) the desire of the Muslim community to extend Sharia’s law to all economic activities” (p.2). Therefore, estimating “the efficiency of Islamic banks is an important issue for researchers and regulators” [16, p. 1].

Another strand of empirical literature has focused specifically on the performance and efficiency of Islamic banks during the COVID-19 pandemic. For example, Le et al. [17] investigates the relationship between diversification and Islamic banking system’ performance under the impact of the COVID-19 turmoil and report a positive association. Ashraf et al. [18] using data from the GCC member states, find that stock market investors have not assessed the Islamic banks to be superior to conventional ones during the Covid-19 market meltdown, whereas [19] investigate banks’ income and stock prices respond to the COVID-19 policy measures in countries with the dual-banking system; Their results show that the Shariah compliance does not limit the adverse impact of the COVID-19 crisis on Islamic banking. Finally, Boubaker et al. [20] evaluate the performance and efficiency of 49 Islamic banks across 10 countries during 2019–2020 to assess how those banks can preserve their performance and remain resilient in the aftermath of the COVID-19 pandemic. Most of these studies have reached the conclusion that IBs are more resilient to COVID-19 than their conventional peers. A number of papers have also focused on the performance of banks in the Gulf Cooperation Council (GCC) countries during the COVID-129 outbreak. The findings reveal that GCC banks were negatively affected by the pandemic. However, Islamic banks have performed better than conventional banks [21].

Our study is important for advancing existing research on the MENA region in a few aspects. First, previous empirical studies provide inconclusive evidence on how banking market concentration affects the efficiency of different types of banking systems operating in the MENA region. To fill this gap, we focus our analysis on Islamic (IBs) and conventional banks (CBs), as well as banks with different ownership structures. While the role of different banking systems to the overall banking sector stability has been widely investigated, the differences in bank ownership structure have received much less attention. Therefore, our study is the first one to investigate the relationship between market concentration and efficiency across banks with different structure (i.e., state and foreign-owned banks). Second, we extend previous studies by incorporating the COVID-19 pandemic effect in our analysis. The analysis of different types of banking systems that co-existing in the MENA region (Islamic and conventional) indicates the market concentration effect is more pronounced when the banking institution is Islamic and during the COVID-19 outbreak.

We perform our analysis using a panel dataset that covers data from 225 banks (both Islamic and conventional) in 18 MENA countries over 15 years (2006–2020). Following Sufian [22], we adopt fixed effect model specifications that include year- and country-fixed effects. For robustness purposes, we use the two-step Generalized Method of Moments (GMM) estimation technique that generates comparable results. We find that banks can increase their efficiency when operating in concentrated markets. Moreover, banks in the MENA region experience a nonlinear (inverted) relationship between market concentration and efficiency. In other words, any increase in market concentration, in the case of low and high concentrated markets, will lead to less improvement in bank efficiency than in moderately concentrated markets such as the MENA countries.

We contribute to the existing empirical literature in several ways. *First*, prior studies that focus on banking sector stability have highlighted the effect of market concentration on bank performance and risk. However, previous research is silent about the impact of banking market structure on bank efficiency, specifically, during the COVID-19 outbreak. Therefore, this paper highlights the importance and the impact of the COVID-19 crisis on bank efficiency in the MENA region. We find a positive influence of market concentration on bank efficiency. This effect is more pronounced during the COVID-19 outbreak and when the banking institution is Islamic. The better performance of Islamic banking institutions during the initial stage of pandemic further increases their efficiency, which supports the [23] findings. The positive association of bank profitability with efficiency (specifically, of Islamic banks) provides strong incentives for managers to improve bank operations’ profitability to increase efficiency and for policymakers to device policies that support domestic Islamic banks.

*Second*, the efficiency of banking institutions in the MENA region is often compared between different banking systems that co-exist in this region [16, 23]. However, the impact of banking market concentration on the efficiency level of these banks (Islamic and conventional) has not been investigated. Therefore, we present a new approach to analyzing market concentration’s impact on bank efficiency. Specifically, we use two-dimensional approach: first, we analyze this effect across the two banking systems (Islamic vs. conventional), and then, we compare banks with different ownership structure (government-owned vs. foreign-owned banks). Our analysis indicates that the market concentration effect is strongly significant and positive for the government-owned banks but irrelevant to the group of foreign-owned banks. Moreover, the concentration effect is more pronounced for domestic Islamic banking institutions. This result somewhat supports [24]. in their study of Malaysian banks that the technical efficiency and scale efficiency of domestic IBs are higher. In comparison, foreign IBs operate at higher pure technical efficiency.

*Third*, we extend the findings of earlier research on the determinants of bank efficiency in the context of the MENA region. For example, Kablan and Yousfi [25] analyzed Islamic banks’ efficiency over the period of 2001–2008 and reported that market power and profitability have negative impact on the efficiency of Islamic institutions. However, concentration leads to higher costs through slacks and inefficiency. In contrast, our study finds that profitability (among other bank-specific characteristics such as deposits, size, and liquid assets) is positively correlated with Islamic banks’ efficiency. This result aligns with Sufian and Noor [26], who analyze the efficiency of IBs in the MENA and Asian countries during the 2001–2006 period and suggest that profitability (among other bank-specific characteristics such as loans, size, and capitalization) is positively correlated with bank efficiency. We also support Rashid and Jabeen [27] who report that “operating efficiency, deposits, and market concentration are significant in explaining performance of Islamic banks” (p. 1). Our findings are important for bank managers who should formulate appropriate strategies to promote bank profitability and increase efficiency to overcome the negative impact of the COVID-19 pandemic.

Our paper is organized as follows: Section 2 contains the literature review and the main hypotheses related to the concentration/efficiency-performance relationship. Section 3 describes the data set and the empirical model. Section 4 provides a detailed analysis of the empirical analysis results and their interpretations. Section 5 includes a robustness check and alternative specifications. Finally, Section 6 presents our conclusions and the main recommendations.

## Theoretical background and hypotheses

### Market structure and bank efficiency hypotheses

There is no doubt that the COVID-19 pandemic and the subsequent economic crisis have changed banks’ profits and risk sources, forcing them to change their business model [28]. According to Kozak and Wierzbowska [1], the expected increase in banking market consolidation due to the recent global crisis (COVID-19 pandemic) will most likely affect banks’ efficiency and ability to finance economic development. Moreover, Hamadi and Awdeh [29] concluded that “per se is not harmful to financial development. Nevertheless, concentration combined with bank market power may deteriorate the development of MENA financial systems” (p. 1). Since the COVID-19 has had a significant impact on the stability of banking systems in the MENA region, investigating the effect of concentration on bank efficiency is an important research question. However, the existing literature fails to provide a robust explanation of how market structure impacts bank efficiency in times of crisis such as COVID-19 pandemic.

According to the theoretical literature, three hypotheses describe this relationship: the quiet life hypothesis, the efficient structure hypothesis, and the information generation hypothesis. The quiet life (QL) hypothesis developed by [30], postulates that firms possessing market power, instead of extracting rents in a concentrated market, make an inefficient allocation that negatively affects their performance. According to Kozak and Wierzbowska [1], they “do not make enough efforts to improve the quality of products and operations management, which leads to a drop in efficiency” (p. 40). This hypothesis aligns with the structure-conduct-performance (SCP) paradigm, which postulates that “high market concentration and market power motivate banks to set favorable prices and achieve higher and extraordinary income” [1, p. 40]. Although this may incentivize banks to increase their efficiency by cutting costs, intensive competition is regarded to be detrimental to the banks’ financial performance due to the loss of market power. If competition is believed to enhance efficiency (QL hypothesis), the importance of considering the banks’ level of efficiency is high since banks with larger market power could choose the quiet life and reduce their cost efficiency. According to Kozak and Wierzbowska [1], the earlier research has proved that, in the short term, banks with a substantial market share increase prices and generate higher profits but, in the long run, “their improper loans monitoring, and excessive operating costs lead to a drop in their efficiency and competitiveness” (p. 40). In the subsequent years, these findings have been further confirmed by a number of relevant studies (see [4, 31–35] among others).

The efficient structure (ES) hypothesis, which was proposed by [36], predicts that “under the pressure of market competition, more efficient firms acquire less efficient competitors and then, due to the use of economies of scale and scope, reduce operating costs, diversify the product offering and increase profits” [1, p. 40]. Thus, they obtain a favorable position in the market. This process increases market concentration but also improves the efficiency of firms operating in it. The ES hypothesis also applies to the banking sector [3, 37–39]. In this case it assumes that “a market becomes more efficient as it becomes more concentrated” [5, p. 1]. The ES hypothesis is consistent with the relative market power (RMP) paradigm, which predicts that “larger firms, due to greater economies of scale and scope, have the capacity to decrease operating costs and to expand the diversification of their products, and hence, increase the efficiency” [1, p. 40]. According to [5, 40], such activities can be performed by banks irrespective of the market concentration level in the banking sector.

The information generation (IG) hypothesis was applied to the banking sector for the first time [41]. According to IG assumptions, “there is a negative association between competition and efficiency which suggests that banks place more emphasis on quantity rather than the quality of assets in order to achieve larger market share” [1, p. 40]. Moreover, “the more competitive the banking market becomes, the more customers are prompted to switch between banks, decreasing their motivation to collect additional information about their clients. As a result, the quality of loan portfolio deteriorates, and so does the efficiency of banks’ operations” (p. 40). Conversely, higher banking market concentration and less aggressive competition encourage banks to invest more in both collecting soft information on their customers and better credit monitoring practices; this contributes to the enhancement of credit portfolio quality and the improvement of bank operations’ efficiency [42].

These hypotheses have been tested in the empirical literature and reached different results depending on the region or country selected. For example, using a sample of six transition countries of South-Eastern Europe over the period 1998–2008, Fang et al. [43] find that the degree of individual banks’ competitiveness positively correlates with bank efficiency. Kasman and Carvallo [44] have analyzed the cost and revenue efficiency of banks in 15 Latin American and Caribbean countries and reported that banking market structure is positively related to efficiency and that competition enforces the banks to be more cost-efficient. Homma [5] tested the ES hypothesis for large banks in Japan from 1974 till 2005, and observed that market concentration reduces bank efficiency, which supports the QL hypothesis. Consistent with the ES hypothesis, the authors find that more efficient banks become larger. Finally, Otero et al. [23] analyzed cost efficiency and its determinants in the MENA countries during the period of 2005–2012. The results indicate that cost efficiency and bank performance are positively related. In contrast, the relationship between the level of concentration and market share is negative thus supporting the QL hypothesis. Building on these arguments and the existing theoretical and empirical evidence discussed above, we test the following hypotheses.

As mentioned above, the quiet life (QL) hypothesis suggests that banks, instead of extracting rents in a concentrated market, make an inefficient allocation that negatively affects their performance [1]. Thus, under this hypothesis, “a negative relationship between efficiency and market structure is established; in other words, a higher level of efficiency can be found in markets with low concentration and for firms with a small market share” [23, p. 7]. In support of this hypothesis, Berger and Hannan [45] find that banks in more concentrated markets are less cost-efficient than those with more competitors. The existing evidence from the MENA region is, however, scarce. For example, Otero et al. [23] find that “the level of concentration and market share have a negative influence on technical efficiency, thus supporting the QL hypothesis” (p. 1). Using an extended period of observation (2006–2020) and a larger number of banks from 18 MENA countries, we test the predictions of the QL hypothesis as follows:

*Hypothesis 1*: *Market concentration is negatively associated with bank efficiency*.

The results of previous studies confirm the existence of a strong correlation between banking market concentration and bank efficiency. According to the empirical literature, market concentration could affect bank efficiency either positively or negatively [1]. The first view would provide evidence in support of the ES hypothesis, which assumes that more efficient banks take over their competitors and increase the market concentration. The second view would prove the QL hypothesis, which assumes that increase in concentration decreases banks’ motivation to collect additional information about their customers and to monitor borrowers, which, in turn, leads to a decrease in efficiency. Previous research also reports the existence of a U-shaped relationship between competition and bank risk-taking [46, 47]. Likewise, Kozak and Gwiazdowsk [1] report a non-linear (inverted) relation between banking market concentration and efficiency. Based on these findings, we test the following hypothesis:

*Hypothesis 2*: *There is a nonlinear (inverted) association between market concentration and bank efficiency in the MENA region*.

Prior studies on the MENA region have documented the dominance of IBs over CBs in terms of higher levels of pre-crisis efficiency. This has determined the superior stock performance of Islamic banking institutions during the outbreak of the COVID-19 crisis [48]. Furthermore, the market concentration effect on bank efficiency could be different between CBs and IBs due to the different business models and risk management practices applied by Islamic and conventional banking institutions [49]. The expected increase in banking market consolidation due to the recent global crisis (COVID-19 pandemic) will most likely affect bank efficiency and their ability to finance economic development as observed in other developing regions (see e.g., [1]). As a result, we may expect that the COVID-19 crisis will intensify the effect of market concentration on bank efficiency. Based on these assumptions, we test the following hypotheses:

*Hypothesis 3a*: *The effect of concentration on bank efficiency is expected to be different between Islamic and conventional banks*.
*Hypothesis 3b*: *The effect of concentration is expected to be more pronounced during the COVID-19 outbreak*.

According to Kozak and Gwiazdowsk [1] statement, “the impact of banking market concentration on efficiency might vary depending on the ownership structure. In general, foreign-owned banks in developing countries are considered to be both better managed and/or technologically equipped than domestic-owned banks, and thus achieve higher profit efficiency and a better competitive position” (p. 46). Previous research provides conflicting evidence on this issue. For example, Fries and Taci [50] investigated banks in 15 Eastern European countries and concluded that privatized banks with prevailing foreign ownership are more cost-efficient than their state-owned competitors. In contrast, [1] report that banking market concentration in the SECE region similarly influences domestic-owned and foreign-owned banks’ efficiency. Since no evidence existed for the banking systems in the MENA region, we test the hypothesis that the concentration effect could be different across government-owned and foreign-owned banks.

*Hypothesis 4*: *There is a differential effect of market concentration on the efficiency of government- and foreign-owned banks in the MENA region*.

### The determinants of bank efficiency

In addition to banking market concentration, prior studies evaluate certain bank efficiency determinants. As one of the most common factors included in the research analysis, size shows mixed results. For example, Ahmad [51] evaluates the cost and profit efficiency of 20 banks in Jordan from 1990 to 1996 using both non-parametric (DEA) and parametric (SFA) techniques. The results reveal that larger banks are more profit-efficient than others. Likewise, Limam [52] investigates the technical efficiency of eight banks in Kuwait from 1994 to 1999 and finds that the larger the bank, the better the efficiency. Chen et al. [53] estimate cost, technical and allocative efficiency of 43 Chinese banks from 1993 to 2000, and report that large and small banks perform better than medium-sized ones. However, Darrat et al. [54] using a sample of 8 banks operating in Kuwait, find that the smaller banks are more competent than the larger ones. Finally, Sarsour and Daoud [55] point out that large banks have lower cost efficiency than their smaller counterparts, indicating the presence of diseconomies of scale for the investigated Palestinian banks. The size of bank only matters in case of Islamic banks for enhancement of efficiencies [13].

According to Kozak and Gwiazdowsk [1], the “magnitude of the impact of competitive conditions on banks’ performance varies significantly across countries and depends on bank ownership” (p. 40). Prior research reports conflicting results. For example, Weill [56] reports that foreign banks in transition countries are more technically efficient than domestic ones. This result aligns with [57], who find that foreign ownership is associated with higher bank efficiency, and [58] claiming that foreign presence improves bank efficiency, primarily in countries with high financial freedom. However, Al-Tamimi and Hussein [59] who examine bank performance in the United Arab Emirates (UAE) from 1997 to 2001, report that most UAE commercial banks are poorly management and domestic banks are better organized than foreign ones. Likewise, Turk [60] examines cost efficiency in Lebanese banks following a period of deregulation and discovers that domestic banks are as efficient as their foreign rivals. Finally, Dong [61] investigates Chinese banks’ cost efficiency from 1994 to 2007 and finds that “both state-owned banks and foreign ones are more efficient than their domestic private counterparts, and larger banks tend to be relatively more efficient than smaller banks” (p. 1). Previous studies also find that individual bank characteristics such as cost-effectiveness, capitalization, liquidity, and profitability are also important determinants of bank efficiency [1, 26, 62]. We include some of these variables in our analysis as control variables.

Based on the analysis of the existing empirical literature, we reached the following conclusion. Although previous studies have examined the impact of market structure on bank efficiency in different countries and/or regions, they have some limitations. *First*, many of these studies refer to the banking sectors in the developed countries of North America [33], Japan [5] and Western Europe [34]. Thus, the findings of these studies arguably limit their generalization to MENA countries because there are substantial differences in terms of financial, political, regulative, and economic features, among others, between developed and developing countries and even within the MENA region [13]. *Second*, most studies that focus on banking sector stability have highlighted the effect of market concentration on bank performance and risk. However, previous research is silent about the impact of banking market structure on bank efficiency, specifically, during the COVID-19 outbreak. *Third*, the efficiency of banking institutions in the MENA region is often compared between different banking systems that co-exist in this region [16, 23]. However, the impact of banking market concentration on the efficiency level of these banks (Islamic and conventional) has not been investigated. Moreover, the policy implications of a future consolidation in the banking sectors in the MENA region are also unknown.

Finally, as the pandemic has accelerated the shift towards digital banking, especially during the lockdown and social distancing, and enhanced the demand for online banking, CBs were required to invest heavily in digital infrastructure to keep up with this demand [63, 64] and diversify their product portfolio to increase their market share. With the concern of their societal impact, IBs are also suggested to invest in digital platforms to achieve digital financial inclusion and increase financial stability. We expect these recent developments (Fintech transformation, digitalization) to have a positive impact on banks’ efficiency and the level of their market competitiveness. This, in turn, will release the pressure of market competition and the need for further bank consolidation caused by the COVID-19 pandemic.

## Data and methods

### Sample selection

This study uses panel data for the MENA banks over a period of 15 years (2006–2020) and builds on the dataset of [65]. The financial data used in their study are collected from the database of Orbis Bank Focus (Bureau Van Dijk), together with the annual reports of the banks included in the sample. S1 Table supporting information describes all the variables used in the analysis and their sources. Summary statistics for the number of banks in each country, the number of Islamic and conventional banks (see S1 Data), and the number of government-owned and foreign-owned banks in presented in Table 1. The data set contains 3,375 bank-year observations for the various financial variables used in this study. To alleviate the possible distortion on the estimation results, bank-level explanatory variables are winsorised at the levels of 1% and 99%.

**Table 1:** Composition of banks by country.

Country/Banks	Total	Conventional	Islamic	Government ownership	Foreign ownership	Average no. of years of obs.	Total Obs.
Algeria	9	9	0	1	4	10.25	135
Bahrain	20	9	11	2	11	12.05	300
Egypt	25	23	2	7	10	12.11	375
Iran (Islamic Republic of)	11	0	11	0	2	10.45	165
Iraq	7	5	2	0	3	11.31	105
Israel	8	8	0	3	2	12.50	120
Jordan	15	12	3	7	11	12.43	225
Kuwait	11	5	6	5	4	12.75	165
Lebanon	23	22	1	1	5	10.27	345
Morocco	8	8	0	3	4	11.62	120
Oman	7	6	1	3	3	12.67	105
Palestinian Territory	4	2	2	3	0	13.00	60
Qatar	10	6	4	5	2	13.18	150
Saudi Arabia	13	8	5	2	3	11.99	195
Syrian Arab Rep.	12	9	3	0	9	12.90	180
Tunisia	13	11	2	1	6	12.13	195
United Arab Emirates	25	16	9	3	9	12.22	375
Yemen	4	3	1	0	0	10.14	60
**Total**	**225**	**162**	**63**	46	88	11.89	3,375
In percentage	100.00	72.00	28.00	20.44	39.11		

The total sample includes 225 banks in 18 countries in the MENA region. The analysis uses country-specific and bank-level data over a period of 15 years (2006–2020). The table shows the total number of banks, the number of conventional banks (CBs) and Islamic banks (IBs) per county, the number of listed and unlisted banks, and the average number of years of observation. The last column represents the total number of observations per country.

### Empirical specification

We explore the issue of whether banking market concentration has a significant impact on bank efficiency and if there is a significant effect of the COVID-19 outbreak on this relationship. Therefore, we estimate the following model:

ESit=ϑ0+ϑ1HHIit+ϑ2HHIit^2+ϑ3HHI*IBit+φXit−1+ωDt+εit (1)

ESit=ϑ0+ϑ1CR3+ϑ2CR3it^2+ϑ3C3*IBit+φXit−1+ωDt+εit (2)

In models (1) and (2), *ES*_*it*_ is the estimated value of the efficiency score for bank *i* in year *t*. Banking market concentration is proxied by the Herfindahl-Hirschman index (HH-index) where the index is a measure of market concentration calculated as the sum of the squared market shares for each bank in a country. Our second measure is the concentration ratio computed as the share of top three banks in total assets of a country’s banking sector (CR3). Moreover, we use an alternative measure of market concentration computed as the share of top five banks in total assets of a country’s banking sector (CR5). Guided by previous literature, we define *X*_*it-1*_ as a vector of bank-specific (or country-specific) variables that are identified as determinants of bank efficiency. The vector of dummy variables (*D*_*t*_) includes IB_it_ variable that equals 1 if a bank is Islamic and 0 otherwise, and Crisis time dummy (COVID-19_it_) that takes the value of 1 for the year 2020, and 0 otherwise. We also control for the country- and year- fixed effects in each model. Next, we assess if the concentration effect is more pronounced in the sample of IBs; therefore, we introduce in Eqs 1 and 2 an interaction term between the concentration measure and the IB dummy variable. Likewise, we test the moderating effect of COVID-19 crisis on bank efficiency for each group of banking institutions (IBs and CBs). All the variables in Eqs (1) and (2) are described in supporting information.

We use fixed effect/random effect specifications and perform a Hausman test where the null hypothesis is that the preferred model is random effects vs. the alternative fixed effects. The choice between random and fixed effects specification depends on the Prob>chi^2 being more or less than 5%, respectively. In order to avoid omitted-variable bias, we control for a vector of bank-specific characteristics and general economic conditions that may affect bank efficiency. Moreover, we introduce in our baseline model additional bank-level variables (e.g., ROA, ROE, and C/I) since they are reported to be conducive to bank [1, 66]. For robustness purposes, we re-estimate Eqs (1) and (2) with a two-step system GMM estimator (Generalized Method of Moments or GMM estimator). This method helps to overcome the endogeneity problem that there is no clear distinction between what independent and dependent variables [67]. The validity of the instruments is tested using a Sargan-Hansen test of over-identifying restrictions, while the AR(1) and AR(2) tests detect the first- and second-order autocorrelation in first differences. The correlation matrix (not presented here to conserve a space) indicates the lack of significant correlation between the dependent and independent variables used in our analysis. The results for the variance inflation factor (VIF) also suggest that our estimation results do not experience any multicollinearity issues.

### Dependent variable

Our dependent variable is bank efficiency. We follow Mateev and Nasr [65] and estimate bank efficiency by using the Data Envelopment Analysis (DEA), which was first proposed by [68]. According to Mirzae et al. [48], mathematically, “DEA is a linear programming-based methodology for benchmarking a set of decision-making units (DMUs) such as a bank with multi-inputs and multi-outputs. DEA estimates the production possibility frontier and evaluates the efficiency of each DMU against the frontier” (p. 21). DEA is a widely used approach to determine the efficiency in the banking sector. For example, Aghimien et al. [69] investigates the efficiency level of Gulf Cooperation Council (GCC) banks on technical efficiency (TE), pure technical efficiency (PTE) and scale efficiency (SE). The results of the study indicate that many GCC banks are operating within an optimal scale of efficiency. Furthermore, the findings show managerial inefficiency in the use of resources. Hussain et al. [70] employs the Data Envelopment Analysis (DEA) approach to examine the microfinance institutions (MFIs) efficiency levels using data for from six Asian countries. The study has important policy implications since governments or policymakers can establish effective national policies and strategies related to MFIs.

DEA can be either input- or output-orientated. Under constant returns to scale (CRS) both input- and output- oriented DEA models produce the same efficiency scores, while under variable returns to scale (VRS), the efficiency scores in two models may be different. The reason is that “input-oriented DEA method defines the frontier by seeking the maximum possible proportional reduction in input usage, with output levels held constant, for each DMU” [48, p. 21]. While, for the output-orientated case, the DEA method seeks the maximum proportional increase in output production, with input levels held fixed. Formally, our model for DMUs can be presented as follows (see [4,8] for more details):

**Table:** 

CRS Model	VRS Model
Max h *λ*, h, S ^-^_I_, S ^+^_r_ s. t. ∑*_j_λ_j_*x*_ij_* + S^-^_I_ = X_ij0_ Ɐ_i_ ∑*_j_λ_j_*y_rj_ – S^+^_r_ = hy_rj0_ Ɐ_r_ S ^-^_I_, S ^+^_I_ ≥0 Ɐ_i,_ Ɐ_r_ *λ_j_* ≥ 0 Ɐ*_j_*	Max h *λ*, h, S ^-^_I_, S ^+^_r_ s. t. ∑*_j_λ_j_*x*_ij_* + S^-^_I_ = X_ij0_ Ɐ_i_ ∑*_j_λ_j_*Y_rj_ – S^+^_r_ = hy_ij0_ Ɐ_rb_ ∑*_j_λ_j_* = 1 S ^-^_I_, S ^+^_I_ ≥ 0 Ɐ_i,_ Ɐ_r_ *λ_j_* ≥ 0 Ɐ_j_

Where, S_i_ and S_r_ represent slack variables and *j* represents bank number.

The DEA scores are computed by country, year, and bank type, for the period 2006–2020 (S1 Data). Supporting information presents summary statistics of DEA’s input and output variables for each group of banks (CBs and IBs). S3 Table shows a diversified structure of bank efficiency across different MENA countries included in our sample (see S3 Table). The most efficient banks operate in Morocco (76%), Saudi Arabia (68.6%), and Qatar (64.1%), and the least efficient are in Jordan and Yemen (around 40%. The average efficiency in the MENA region equals 53.4%. Moving onto the efficiency of IBs compared with CBs with reference to the constant returns-to-scale (CRS) scores, the first type of bank is, on average, not better than the second one (an efficiency score of around 55%). We also compare the efficiency between the two banking systems using the other two DEA measures: variable return-to-scale (VRS) and returns-to-scale (SCALE). We observe that the average efficiency across the three DEA scores for the whole sample period is slightly higher for CBs than IBs (68.1% vs. 67.9%). The results are available on request.

### Independent and control variables

To measure the level of banking market concentration level we use the HH-index (S1 Data). The HH-index is a measure of market concentration computed “by summing the squares of the market shares of every bank in the market or a country, and it varies between zero (situation of pure and perfect competition) and 10,000 (100^2: monopoly position)” [71, p. 35]. We create an interaction term between the HH-index and the Islamic dummy variable to estimate if the impact of banking market concentration is more pronounced in the sample of Islamic banking institutions. Market concentration could affect bank efficiency either positively or negatively. The first option aligns with the efficient structure (ES) hypothesis, which assumes a positive correlation between concentration and efficiency. In contrast, the second option would prove the quiet life (QL) hypothesis, which assumes that an increase in concentration leads to a drop in efficiency. As an second indicator of market concentration, we use the concentration ratio computed as the share of the top three banks in total assets of a country’s banking sector (CR3). Following a quadratic term of concentration variable is used in Eq (1) an Eq (2) to account for the potential nonlinear relation between concentration and efficiency.

Guided by previous studies [1, 16, 23], we employ several bank-level characteristics that the empirical literature reports as significant bank efficiency determinants. These include deposits, loans, liquid assets, loan growth rate, size, and equity to total assets ratio. They are employed in the regression analysis as control variables. Prior studies identify size as one of the most commonly used determinants of bank efficiency; however, the results are mixed. Some studies argue that large banks are more cost-efficient than small ones because the former has the ability to increase their revenue with fewer costs [72]. Others point out that large banks have lower cost efficiency than their smaller counterparts, which indicates the presence of diseconomies of scale [55]. In line with [1, 23], we assume a positive relationship between bank size and efficiency. Following [73, 74], the leverage ratio (equity to total assets) is included to control for differences in the risk preferences across banks. The liquidity ratio (liquid assets to total assets) is used as an indicator that “banks with more liquid assets are in a better position to reduce their balance sheet and to cope with financing difficulties” [75, p. 10].

Next, we employ a few measures of bank profitability. Empirical literature reports a positive impact of bank profitability on efficiency. According to Kozak and Gwiazdowsk [1], “generating higher profits from total assets or total equity and lowering operating costs increase a bank’s efficiency” (p. 43). For this reason, we introduce the following measures of banks’ profitability and effectiveness: Return on assets (ROA), Return on equity (ROE), and Cost-to-Income ratio (C/I), and assume a positive relationship between profitability measures and bank efficiency. In addition, following Kaufmann et al. [76] approach, we create an index, institution, which is the mean of the six variables for each country in the sample (see S1 Table for a short description of these variables). A higher value of the index indicates a better institutional environment in the sample country. To control for potential differences in bank’s external environment, we applied the following macroeconomic variables: real GDP growth and inflation rate. We may expect that higher GDP growth contributes to improved efficiency, while inflation increases bad debts and, therefore, makes banks bear additional costs to cope with credit risk, which increases their inefficiency [1, 77].

## Results and discussions

### Descriptive statistics and sample banks

We investigate the impact of banking market concentration on bank efficiency. We also address the question of whether this impact is significantly different between IBs and CBs. For this purpose, in Tables 2 and 3 we present the descriptive statistics on bank efficiency (DEA scores) and different bank-level and country-specific explanatory variables for each group of banks (Islamic and conventional).

**Table 2:** Descriptive statistics of DEA’s input and output variables for CBs and IBs.

	All				IB (1)				CB (2)				
Variable	Mean	Std. Dev.	Min	Max	Mean	Std. Dev.	Min	Max	Mean	Std. Dev.	Min	Max	Mean test (*t*-stat)
**Inputs (% of total assets)**													(1)—(2)
Staff costs	0.007	0.012	-0.010	0.160	0.004	0.006	-0.010	0.030	0.007	0.013	0.000	0.160	1.000
Fixed assets	0.029	0.057	0.000	0.750	0.027	0.061	0.000	0.750	0.030	0.056	0.000	0.890	-0.510
Deposits	0.578	0.331	0.000	0.960	0.690	0.277	0.000	0.960	0.556	0.337	0.000	0.970	5.830***
Impaired loans	0.014	0.019	-0.010	0.210	0.010	0.007	-0.010	0.050	0.014	0.020	0.000	0.210	2.375***
**Outputs (% of total assets)**													
Loans	0.424	0.279	0.000	0.990	0.478	0.229	0.000	0.930	0.413	0.286	0.000	0.990	1.000
Other earning assets	0.345	0.251	0.000	1.000	0.249	0.189	0.000	0.990	0.364	0.257	0.000	1.000	-0.510
Non-interest income	0.013	0.031	-0.030	0.480	0.006	0.009	-0.010	0.120	0.014	0.034	-0.030	0.480	5.830***

**Table 3:** Descriptive statistics of sample banks (CBs and IBs).

	All Banks				Conventional Banks				Islamic Banks				*Mean test*
	*# obs*	*Mean*	*Median*	*SD*	*# obs*	*Mean*	*Median*	*SD*	*# obs*	*Mean*	*Median*	*SD*	*p-value*
***Efficiency measures (DEA)***													
*ES 1 (CRS)*	3347	0.52	0.48	0.25	2430	0.51	0.48	0.25	917	0.52	0.49	0.26	0.000***
*ES 2 (VRS*	3347	0.86	0.91	0.17	2430	0.87	0.91	0.17	917	0.84	0.88	0.16	0.000***
*ES 3 (SCALE)*	3347	0.58	0.57	0.24	2430	0.58	0.56	0.24	917	0.60	0.59	0.24	0.000***
***Profitability and Cost efficiency measures***													
*Return on Assets (ROA)*	3324	1.2%	1.2%	2.8%	2268	1.4%	1.2%	1.9%	856	0.9%	1.2%	4.3%	0.000***
*Return on Equity (ROE)*	3324	10.0%	10.7%	30.4%	2268	9.8%	11.0%	29.5%	856	10.6%	9.6%	32.9%	0.000***
*Cost-to-income ratio (C/I)*	3347	49.7%	45.9%	24.4%	2430	48.3%	45.1%	21.5%	917	52.8%	46.8%	30.2%	0.075*
***Market Concentration***													
HH-index	3347	0.25	0.19	0.18	2430	0.25	0.19	0.17	917	0.26	0.18	0.21	0.352
Concentration ratio (CR3)	3347	0.65	0.60	0.19	2430	0.66	0.62	0.19	917	0.64	0.58	0.18	0.610
***Bank-level characteristics***													
*Deposit/ Total Assets*	3347	76.6%	80.4%	15.0%	2430	78.0%	80.6%	11.7%	917	72.2%	79.6%	21.6%	0.000***
*Loan/ Total Assets*	3347	49.0%	53.2%	20.4%	2430	48.5%	50.9%	19.7%	917	50.4%	57.5%	22.2%	0.027**
*Size*	3347	5.35	4.99	1.90	2430	5.36	4.97	1.91	917	5.30	5.09	1.89	0.350
*Loan Growth*	3347	-2.7%	5.0%	39.0%	2430	7.8%	6.6%	25.5%	917	-18.2%	2.0%	49.1%	0.120
*Liquid assets*	3347	25.7%	21.7%	17.9%	2430	26.3%	22.0%	17.6%	917	24.3%	20.7%	18.7%	0.000***
*Total Equity/Total Assets*	3347	15.2%	11.6%	14.4%	2430	13.3%	11.4%	8.8%	917	20.7%	12.6%	23.2%	0.000***
***Risk measures***													
*Non-performing loans to GL*	3347	8.2%	4.6%	13.6%	2430	8.0%	4.6%	13.0%	917	9.0%	4.2%	15.1%	0.812
*Log Z*	3347	2.56	2.93	1.33	2430	2.70	3.07	1.30	917	2.13	2.56	1.35	0.000***
***Regulation and institution***													
Institution	238	-0.29	-0.19	0.67	238	-0.31	-0.27	0.66	210	-0.22	-0.09	0.68	0.001***
***Ownership measures***													
*Ownership concentration*	2157	47.8%	40.7%	29.6%	1582	49.2%	42.3%	29.5%	575	44.1%	37.0%	29.7%	0.001***
*Government ownership*	556	22.2%	8.5%	30.7%	428	24.1%	10.0%	32.6%	128	16.0%	7.6%	22.2%	0.000***
*Foreign ownership*	1312	41.6%	34.9%	30%	1008	43.3%	35.4%	30.8%	304	35.8%	30.0%	26.2%	0.000***
***Macroeconomic variables***													
GDP Growth	238	3.7%	3.3%	4.0%	238	3.7%	3.2%	3.8%	210	3.8%	3.5%	4.5%	0.715
Inflation	238	0.0%	-1.5%	11.8%	238	1.2%	-0.8%	11.7%	210	-3.3%	-2.7%	11.3%	0.000***

The sample includes 225 banks in 18 countries in the MENA region. The sample of conventional banks includes 162 banks, and the sample of Islamic banks– 63 financial institutions. As measures of bank profitability, we use Earnings to total assets (EARTA) and Earnings to gross loans (EARGL), and for efficiency the measures are DEA efficiency scores (CRS, VRS and SCAL). Market concentration is measured by the HHI and concentration ratio (CR3). All the variables except market competition indices and institutions are in percentages. Bank characteristics for different groups of banks are computed using data for the period 2006–2020. Bank-level characteristics, regulation, ownership, and macroeconomic variables are described in S1 Table.

The efficiency scores in this study are calculated using the Data Envelopment Analysis (DEA) technique proposed by [68]. Following [48, 78], we use the following variables as inputs: (i) staff costs as a proxy for labor inputs, (ii) fixed assets as a proxy for capital input, (iii) total deposits as a proxy for financial input, and (iv) impaired loans to account for the credit risk. Accordingly, we consider three outputs: (i) gross customer loans; (ii) other earning assets, including investment securities, loans and advances to banks, and other investment; and (iii) non-interest income, including net fees and commissions, net gains/losses on trading and derivatives, and other operating income. Mirzae et al. [48] claim that non-interest income can be viewed as a proxy for off-balance-sheet activities, which is an important component of banking business.

**Fig 1:**
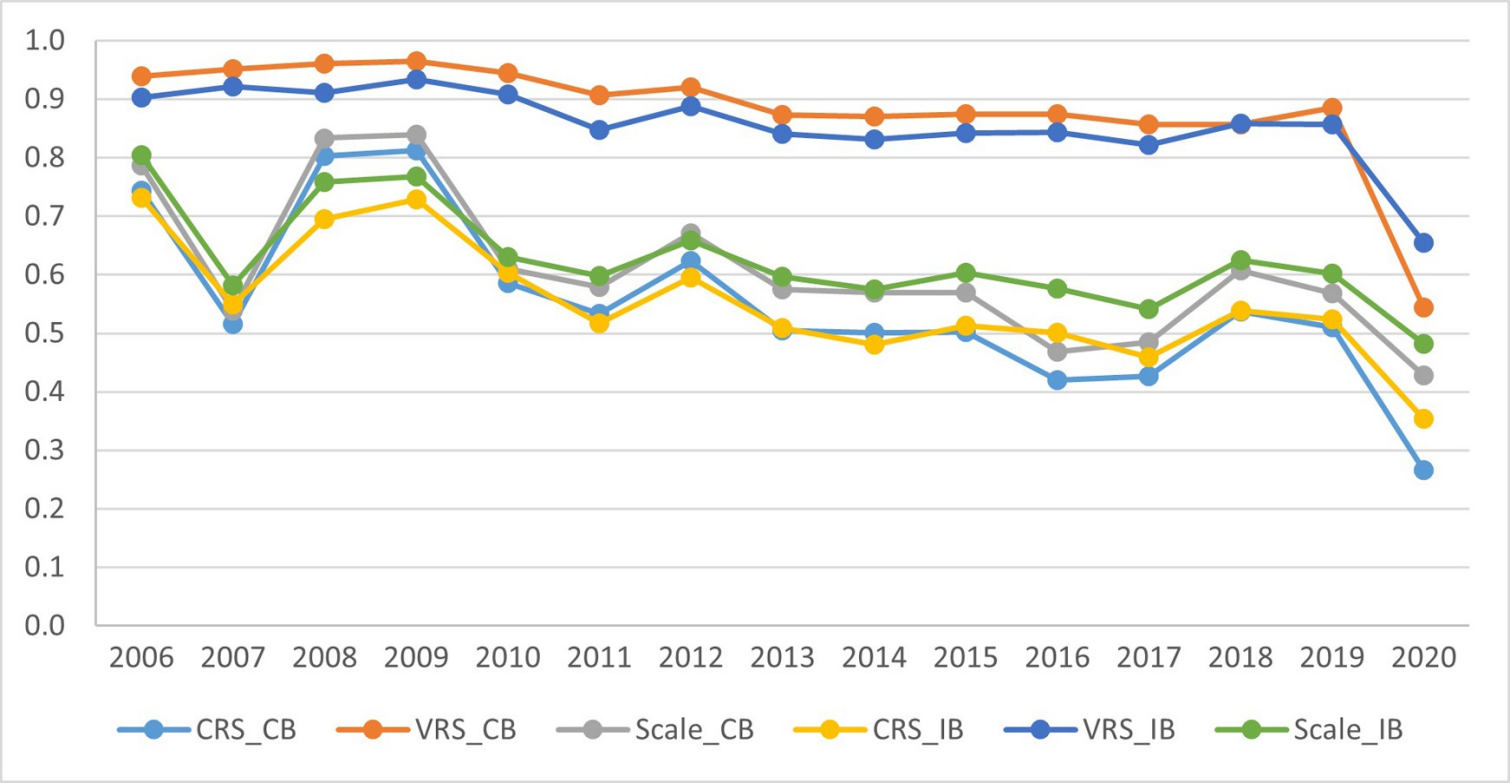
Efficiency scores evolution during the period 2006–2020.

Table 3 provides information for the efficiency scores (CRS, VRS, and SCALE), which were obtained using the non-parametric Data Envelopment Analysis (DEA) for the period of 2006–2020. We observed that the mean differences in the efficiency levels are statistically significant at the 1% significance level. Fig 1 represents different DEA efficiency scores (CRS, VRS, and SCALE) by model specification and type of banks (CBs vs. IBs). The careful examination of the line charts presentation by bank type reveals the dominance of IBs over CBs in terms of efficiency levels (SCALE and CRS); this is especially valid for the period after 2011. Regarding the CRS measure of efficiency, the two types of banks perform similarly. The year 2020 witnessed a significant drop in the efficiency of both CBs and IBs. However, IBs efficiency levels exceed those of CBs in 2020.

Our results indicate that CRS efficiency of the IBs were higher than those of the CBs in the years of 2007 and 2020 (see S3 Table). One possible reason is that IBs are considered more resilient during periods of financial stress because of their strong capital adequacy ratios, which are considerably higher than those of traditional banks [79]. A second reason is the fact that IBs operate in highly regulated environments guided by the Shari’ah principles [80] and banks tend to operate more cautiously. More recent papers also confirm our findings. For example, Ashraf et al. [81] finds that during the COVID-19 period Islamic equity investments (IEIs) exhibit significant excess returns on a nominal and risk-adjusted basis. The evidence suggests that IEIs do provide resistance during extreme market downfalls such as COVID-19. Boubaker et al. [82] investigates the question of whether the Islamic banking business model makes corporate earnings more uncertain and reports that that IB’ returns on assets are significantly more uncertain than conventional banks due to higher operational costs. Consistent with earlier evidence, the study also finds that “Islamic banks generally have fewer non-performing loans than conventional banks” (p. 1) and thus are more stable.

Regarding bank performance, the differences in means between the two groups of banks are statistically insignificant for both profitability measures (ROA and ROE). Therefore, IBs profitability is, on average, not better than CBs profitability. However, IBs have a higher level of cost inefficiency when compared with CBs (52.8% vs. 48.3%). Surprisingly, we did not find a significant difference in the level of banking market concentration as measured by the HH-index or the concentration ratio (CR3). The overall mean value of the HH-index is 0.25, which means the MENA banking market is characterized as moderately concentrated, while the share of the top three banks in total assets of a country’s banking sector is relatively high (65% on average). The analysis shows higher level of concentration (measured by HH-index) for conventional banking system than the Islamic one. The countries with highest level of banking concentration are Jordan, Qatar, and Yemen, and those with the lowest concentration level are Algeria, Iraq, and Tunisia. When the concentration level is measured by CR3, the two markets (IBs and CBs) seem quite similar (66% vs. 64%), so we cannot conclude which banking market is more concentrated. Overall, our results align with [80] who found that IBs are not quite different in behavior or dynamics from CBs and that CBs appear to be the benchmark for IBs.

The analysis of the individual bank-level characteristics indicates that most bank-specific variables are significantly different between CBs and IBs. These include the deposit-to-assets ratio, loans to total assets, the number of liquidity assets, and the level of leverage (equity capital to total assets). For example, we observe a positive loan growth rate for CBs, which turns negative for IBs as well as for the entire observation period (2006–2020). Moreover, CBs and IBs seem quite similar in size (measured by the natural logarithm of the bank’s total assets). However, the mean difference between the two variables is statistically insignificant. Tables 2 and 3 shows that the two banking systems are different regarding risk-taking. On the one side, the Z-score is much higher for CBs than IBs (2.70 vs. 2.13), which is evidence of better financial stability. Conversely, IBs are not quite different from CBs concerning the credit risk level measured by the non-performing loans to gross loans (NPL/GL) ratio. Concerning the macro-economic variables, the positive GDP growth rate (3.7% on average) enables banks to generate higher profits and improve efficiency. However, the two groups of banks experience a different impact of inflation, with turns to be negative for IBs (a median value of -2.7%). Overall, our results support previous studies’ findings on banking systems in the MENA region [23, 49, 83].

### The impact of market concentration on bank efficiency

We estimate a panel data regression with the model specification determined in Eq (1) to test Hypothesis 1. Our dependent variable is bank efficiency. We report the results using the efficiency scores of three different efficiency estimation models (CRS, VRS, and SCALE) for all 225 banks in 18 MENA countries. We run the baseline model separately for each measure, controlling the influence of important macroeconomic variables and bank-level characteristics (see S1 File). The outputs of the regression analysis are reported in Table 4.

**Table 4:** Panel regressions of bank efficiency (All banks, 2006–2020).

	Panel A: HHI			Panel B: CR3		
Efficiency measures	CRS	VRS	SCALE	CRS	VRS	SCALE
	Model 1	Model 2	Model 3	Model 4	Model 5	Model 6
Constant	0.387***	0.630***	0.449***	0.387***	0.631***	0.444***
	(0.000)	(0.0001)	(0.000)	(0.000)	(0.000)	(0.000)
*HHI*	0.134**	0.157*	0.124*			
	(0.036)	(0.064)	(0.071)			
*HHI^2*	-0.136*	-0.187*	-0.134*			
	(0.087)	(0.076)	(0.100)			
*CR3*				0.075	0.074	0.116*
				(0.218)	(0.356)	(0.076)
*CR3^2*				-0.092*	-0.119*	-0.146**
				(0.101)	(0.105)	(0.019)
HHI* *Islamic*	0.499***	0.846***	0.593***			
	(0.000)	(0.000)	(0.000)			
CR3* *Islamic*				0.159***	0.291***	0.195***
				(0.000)	(0.000)	(0.000)
*ROA*	0.002	0.007	0.003	0.002	0.005	0.003
	(0.370)	(0.824)	(0.015)	(0.348)	(0.877)	(0.248)
*C/I* * *100*	0.004	0.004	0.011	0.018	0.030	0.028
	(0.924)	(0.939)	(0.817)	(0.661)	(0.586)	(0.539)
*Deposit/Assets*	-0.037*	-0.041*	-0.038*	-0.020	-0.011	-0.018
	(0.065)	(0.101)	(0.079)	(0.309)	(0.671)	(0.398)
*Loan/Assets*	0.019	0.023	0.017	0.002	0.007	0.002
	(0.363)	(0.427)	(0.456)	(0.914)	(0.794)	(0.948)
*Size* * *10*	0.004	0.003	0.005	-0.002*	-0.003*	-0.002*
	(0.757)	(0.879)	(0.768)	(0.094)	(0.082)	(0.087)
*Loan Growth*	-0.001	-0.002	-0.002	0.006	0.008	0.002
	(0.677)	(0.507)	(0.522)	(0.842)	(0.853)	(0.948)
*Liquid Assets*	0.020	0.002	0.021	0.020	0.005	0.022
	(0.433)	(0.499)	(0.438)	(0.436)	(0.987)	(0.435)
*Equity/TA*	0.006	0.007	0.001	0.013	0.045	0.009
	(0.820)	(0.401)	(0.958)	(0.633)	(0.229)	(0.751)
*GDP Growth*	0.063	0.109	0.055	0.062	0.110	0.058
	(0.475)	(0.349)	(0.559)	(0.484)	(0.344)	(0.542)
*Inflation*	0.032	0.007	0.025	-0.054*	-0.147*	-0.074*
	(0.597)	(0.929)	(0.693)	(0.062)	(0.064)	(0.052)
*Islamic*	-0.106***	-0.171***	-0.106***	-0.066***	-0.104***	-0.059***
	(0.000)	(0.000)	(0.000)	(0.000)	(0.000)	(0.000)
*COVID-19*	-0.008	-0.002	-0.006	-0.090***	-0.151***	-0.107***
	(0.613)	(0.927)	(0.708)	(0.000)	(0.000)	(0.000)
Country Dummy	Yes	Yes	Yes	Yes	Yes	Yes
Year Dummy	Yes	Yes	Yes	Yes	Yes	Yes
Number of Observations	3347	3347	3347	3347	3347	3347
*R*-squared (Overall)	0.3957	0.5200	0.4217	0.4032	0.5205	0.4258

The panel data regressions estimate the relation between bank efficiency and market concertation during the period from 2006 to 2020 while controlling for important bank-level and macroeconomic characteristics. The sample includes 225 banks in 18 countries in the MENA region. Banks included in the sample are either conventional banks or Islamic banks. The efficiency measures are DEA efficiency scores computed using different approaches (constant returns to scale approach CRS; variable returns to scale approach, VRS; and the ratio of CRS score to VRS score, SCALE). Bank profitability and cost efficiency are proxied with accounting measures: Return on Assets (ROA) and Cost-to-Income (C/I) ratio. The HH-Index and the concentration ratio (CR3) are used to measure the level of market concentration. Bank-level characteristics and country-specific variables are computed as of year *t-1*. All the regressions control for year- and country-fixed effects. *, **, and *** indicate statistical significance at the 10%, 5%, and 1% level, respectively. Bank-level characteristics, institution, and macroeconomic variables are described in S1 Table.

According to our first hypothesis (H1), we except a negative relationship between banking market concentration and efficiency. The estimated coefficients of concentration measure (the HH-index) are statistically significant and positive (see Panel A). This result confirms the assumption for a significant impact of banking market structure on bank efficiency. The concentration thus creates favorable conditions for banks to increase their profit efficiency. The observed positive relationship with efficiency contradicts our first hypothesis but supports the prediction of the ES hypothesis. It aligns with the findings for other developing regions (e.g., [1] for the SECE region). The analysis of the signs of the concentration coefficient (positive) and of the square term of concentration measure (negative) suggests an inverted U-shaped relationship between banking market concentration and efficiency. In other words, “an increase in market concentration, in the case of low and high concentrated markets, provides less incentive for improvement of bank efficiency, contrary to the case of moderately concentrated markets” [1, p. 44]. This result confirms out second hypothesis (H2).

Our results complement those of [84] who suggest a standard U-shaped relationship between competition and banks’ risk of failure for MENA banks. According to authors, competition-stability and competition-fragility hypothesis could be applied at the same time for MENA banks. Specifically, “in countries where the level of competition is high (Gulf) the rise in competition increases the probability of default but when the level of competition is low (non-Gulf), the increase in rivalry can be positive in terms of risk-shifting and efficiency” (p. 13). Since high concentration is associated with less competition (the market power of banks is high), in moderately concentrated sectors (e.g., MENA) an additional increase in concentration will bring more improvement in profit efficiency. This means that regulatory authorities concerned with improving financial stability in the MENA region should proceed differently depending on the level of concentration in the market. Increased competition among concentrated banks can increase risk taking and decrease efficiency, while it could be a good practice in less competitive markets.

Surprisingly, the analysis reveals a positive but insignificant association between profitability measure (ROA) and bank efficiency. Thus, we cannot confirm the ES hypothesis’s prediction. Previous research on developing markets (see [1] for the SECE region and [23] for the MENA region) reports that the profit efficiency of banks in these countries is positively related to the increase of bank profitability when measured by the return on assets and return on equity but negatively to the level of cost inefficiency. Since both measures (ROA and C/I) are insignificant in our analysis of the MENA region’s banks, we cannot support previous research that finds the improvements in bank profitability and cost efficiency to positively influence efficiency. Therefore, we run additional tests with ROE and NIM as profitability measures (see S4 Table) and find a positive and significant association only for the net interest margin (NIM) variable.

In Table 4 (Panel B) we report the results of estimating Eq (1) using concentration ratio (CR3) as a second measure of market concentration. Though the estimated coefficients of the CR3 ratio are insignificant, the negative sign of its square term signifies that there is an inverted U-shaped relationship between banking market concentration and efficiency, which again confirms our second hypothesis. Next, we test the hypothesis that the concentration effect on bank efficiency is significantly different between Islamic and conventional banking markets (H3a). Therefore, in line with Eq (2), we introduce an interaction term between the concentration measure (HH-index and CR3) and the Islamic dummy indicator in each regression. The results reported in Panels A and B both indicate a significant differential effect of concentration on IBs efficiency for all three efficiency scores (the estimated coefficients of the interaction term between each concentration measure and the IB dummy variable are strongly statistically significant and positive). We will further investigate this issue in the next section.

Referring to the sign and significance of the control variables’ coefficients, we find marginal evidence for the determinants of bank efficiency (see Table 4). The analysis of bank-specific characteristics that are well known to explain efficiency indicates that only the deposits to assets ratio (in the models using the HH-index) and bank size (in the models using the CR3 ratio) are marginally associated with the level of bank efficiency. Both variables hold signs and magnitude as per our expectations. The negative sign of the deposit ratio indicates decreased efficiency of banks with excessive deposit base, which is typical for conventional banking institutions which raise funds through selling demand deposits that are easily accessible. According to existing research, the size of banking institutions is expected to impact bank efficiency significantly. Indeed, we find that bank size has a strong negative influence on bank efficiency, indicating the presence of diseconomies of scale in the investigated banks. This result aligns with [43] but contradicts [1, 23].

Regarding the country-level variables, macroeconomic conditions have a marginal effect on bank efficiency in the MENA region. Specifically, rising inflation weakens bank efficiency (see Panel B). This finding aligns with [1], who report a similar negative effect on profit efficiency for their sub-sample of EU banks. However, GDP growth is insignificant, which contradicts the assumption that the high dynamics of economic development, expressed by the GDP growth rate, enable banks to generate higher profits and improve profit efficiency [85]. We do not find strong evidence for a significant relation between market concentration (measured by the HH-index) and efficiency during the COVID-19 outbreak. The reason could be that we use only one year of data (2020) of pandemic period and its effect is yet to unfold. However, the concentration effect is strongly negative and significant in the regression models using the concentration ratio (CR3), indicating that banks in the MENA countries experienced low efficiency during the COVID-19 pandemic. However, this effect is significant only in the sample using the concentration ratio. Finally, the negative sign of the IB dummy variable speaks for the low efficiency of Islamic banking institutions when measured relative to their conventional counterparts. This finding coincides with the results of our analysis of efficiency per country and bank type (the results are available upon request).

### The differential effect of concentration on IBs efficiency

This section investigates the differential effect of concentration on IBs efficiency and whether this effect is more pronounced during the COVID-19 outbreak. To the best of our knowledge, no prior evidence exists for the global crisis effect on bank efficiency in the MENA region. Therefore, we run our analysis by splitting the entire sample into two sub-samples—conventional and Islamic banks, respectively. We run the Chow [86] test for the compared coefficients to see whether there is a significant difference between the two sets of coefficients for CBs and IBs. Specifically, we test separately if there is a significant difference between the two groups of banks for each variable of interest. The result for ROA shows that we cannot reject the null hypothesis, that is, there is no significant improvement in fit from running two separate regressions. However, in case of concentration index (HHI and CR3), the null hypothesis is rejected, which justifies our approach of using two separate regressions. The outputs of the regression analysis are reported in Tables 5 and 6 (see S2 and S3 Files).

**Table 5:** Panel regressions of bank efficiency (CBs, 2006–2020).

	Panel A: HHI			Panel B: CR3		
Efficiency measures	CRS	VRS	SCALE	CRS	VRS	SCALE
	Model 1	Model 2	Model 3	Model 4	Model 5	Model 6
Constant	0.436***	0.726***	0.493***	0.445**	0.747***	0.501***
	(0.000)	(0.001)	(0.000)	(0.000)	(0.000)	(0.000)
*HHI*	0.159*	0.210*	0.190**			
	(0.056)	(0.060)	(0.033)			
*HHI^2*	-0.198*	-0.294*	-0.244**			
	(0.077)	(0.050)	(0.042)			
*CR3*				0.071	0.065	0.110
				(0.342)	(0.513)	(0.168)
*CR3^2*				-0.064	-0.070	-0.104
				(0.370)	(0.166)	(0.174)
HHI* COVID-19	0.279*	0.219	0.116			
	(0.062)	(0.272)	(0.467)			
CR3* COVID-19				0.129	0.166	0.116
				(0.130)	(0.145)	(0.204)
*ROA*	0.002	0.004	0.001	0.002	0.004	0.001
	(0.540)	(0.377)	(0.720)	(0.519)	(0.412)	(0.676)
C/I* 10	0.002	0.004	0.003	0.001	0.003	0.002
	(0.614)	(0.498)	(0.526)	(0.685)	(0.540)	(0.567)
*Deposit/Assets*	-0.011	-0.022	-0.017	-0.011	-0.021	-0.017
	(0.646)	(0.522)	(0.527)	(0.648)	(0.542)	(0.538)
*Loan/Assets*	-0.007	-0.007	-0.007	-0.007	-0.008	-0.007
	(0.794)	(0.828)	(0.802)	(0.782)	(0.812)	(0.789)
*Size*	-0.004**	-0.005**	-0.003*	-0.004**	-0.005**	-0.003*
	(0.033)	(0.046)	(0.095)	(0.032)	(0.043)	(0.091)
*Loan Growth*	0.009	0.019	0.017	0.011	0.020	0.019
	(0.723)	(0.599)	(0.542)	(0.679)	(0.566)	(0.496)
*Liquid Assets*	0.020	0.002	0.031	0.021	0.002	0.031
	(0.525)	(0.996)	(0.031)	(0.513)	(0.996)	(0.368)
*Equity/TA*	0.044	0.081	0.042	0.046	0.085	0.044
	(0.203)	(0.081)	(0.255)	(0.186)	(0.069)	(0.235)
*GDP Growth*	0.127	0.113	0.125	0.118	0.115	0.122
	(0.253)	(0.445)	(0.295)	(0.293)	(0.441)	(0.308)
*Inflation*	0.052	-0.017	0.031	0.039	-0.038	0.017
	(0.533)	(0.876)	(0.725)	(0.641)	(0.733)	(0.845)
*COVID-19*	-0.039***	-0.135***	-0.126***	-0.097***	-0.134***	-0.125***
	(0.000)	(0.000)	(0.000)	(0.000)	(0.000)	(0.000)
Country Dummy	Yes	Yes	Yes	Yes	Yes	Yes
Year Dummy	Yes	Yes	Yes	Yes	Yes	Yes
Number of Observations	2430	2430	2430	2430	2430	2430
*R*-squared (Overall)	0.3164	0.4272	0.3400	0.3137	0.4236	0.3386

The panel data regressions estimate the relation between bank efficiency and market concertation over the period from 2006 to 2020 while controlling for important bank-level and macroeconomic characteristics. The sample includes 225 banks in 18 countries in the MENA region. Banks included in the sample are only conventional banks. The efficiency measures are DEA efficiency scores computed using different approaches (constant returns to scale approach CRS; variable returns to scale approach, VRS; and the ratio of CRS score to VRS score, SCALE). Bank profitability and cost efficiency are proxied with accounting measures: Return on Assets (ROA) and Cost-to-Income (C/I) ratio. The HH-Index and the concentration ratio (CR3) are used to measure the level of market concentration. Bank-level characteristics and country-specific variables are computed as of year *t-1*. All the regressions control for year- and country-fixed effects. *, **, and *** indicate statistical significance at the 10%, 5%, and 1% level, respectively. Bank-level characteristics, institution, and macroeconomic variables are described in S1 Table.

**Table 6:** Panel regressions of bank efficiency (IBs, 2006–2020).

	Panel A: HHI			Panel B: CR3		
Efficiency measures	CRS	VRS	SCALE	CRS	VRS	SCALE
	Model 1	Model 2	Model 3	Model 4	Model 5	Model 6
Constant	0.286***	0.431***	0.383***	0.323***	0.429***	0.423***
	(0.002)	(0.000)	(0.000)	(0.000)	(0.212)	(0.000)
*HHI*	0.189*	0.289**	0.146*			
	(0.061)	(0.018)	(0.166)			
*HHI^2*	-0.210*	-0.329**	-0.173*			
	(0.056)	(0.014)	(0.134)			
*CR3*				-0.002	0.111	-0.003
				(0.977)	(0.259)	(0.968)
*CR3^2*				-0.006	-0.077	-0.024
				(0.930)	(0.363)	(0.739)
HHI* COVID-19	-0.076*	-0.156*	-0.077			
	(0056)	(0.055)	(0.269)			
CR3* COVID-19				0.197***	0.171**	0.205***
				(0.002)	(0.029)	(0.002)
*ROA*	0.004**	0.005*	0.007**	0.004*	0.006*	0.007**
	(0.026)	(0.100)	(0.041)	(0.081)	(0.101)	(0.015)
*C/I*	0.034	0.025	0.022	0.036	0.026	0.030
	(0.145)	(0.374)	(0.093)	(0.124)	(0.361)	(0.217)
*Deposit/Assets*	-0.013	0.041	-0.003	-0.007	0.047	0.003
	(0.653)	(0.247)	(0.922)	(0.786)	(0.187)	(0.910)
*Loan/Assets*	0.023	-0.007	0.001	0.022	0.009	0.002
	(0.480)	(0.857)	(0.963)	(0.501)	(0.817)	(0.994)
*Size*	-0.003	-0.002	-0.003	-0.003	-0.002	-0.003
	(0.134)	(0.489)	(0.189)	(0.154)	(0.481)	(0.206)
*Loan Growth*	0.001	0.003	0.003	0.001	0.001	0.001
	(0.591)	(0.910)	(0.911)	(0.642)	(0.953)	(0.955)
*Liquid Assets*	0.004	-0.018	-0.008	-0.005	-0.027	-0.014
	(0.910)	(0.716)	(0.838)	(0.990)	(0.578)	(0.734)
*Equity/TA*	-0.056	-0.074	-0.066	-0.058	-0.073	-0.069
	(0.251)	(0.209)	(0.191)	(0.231)	(0.215)	(0.174)
*GDP Growth*	0.089	0.079	0.052	0.014	0.217	0.003
	(0.457)	(0.140)	(0.678)	(0.910)	(0.149)	(0.979)
*Inflation*	-0.168**	-0.220**	-0.216***	-0.205***	-0.284***	-0.240***
	(0.032)	(0.020)	(0.008)	(0.007)	(0.002)	(0.003)
*COVID-19*	-0.085***	-0.097***	-0.097***	-0.215***	-0.229***	-0.232***
	(0.000)	(0.000)	(0.000)	(0.000)	(0.000)	(0.000)
Country Dummy	Yes	Yes	Yes	Yes	Yes	Yes
Year Dummy	Yes	Yes	Yes	Yes	Yes	Yes
Number of Observations	917	917	917	917	917	917
*R*-squared (Overall)	0.6179	0.7618	0.6766	0.6202	0.7614	0.6786

The panel data regressions estimate the relation between bank efficiency and market concertation over the period from 2006 to 2020 while controlling for important bank-level and macroeconomic characteristics. The sample includes 225 banks in 18 countries in the MENA region. The banks included in the sample are only Islamic banks. The efficiency measures are DEA efficiency scores computed using different approaches (constant returns to scale approach CRS; variable returns to scale approach, VRS; and the ratio of CRS score to VRS score, SCALE). Bank profitability and cost efficiency are proxied with accounting measures: Return on Assets (ROA) and Cost-to-Income (C/I) ratio. The HH-Index and the concentration ratio (CR3) are used to measure the level of market concentration. Bank-level characteristics and country-specific variables are computed as of year *t-1*. All the regressions control for year- and country-fixed effects. *, **, and *** indicate statistical significance at the 10%, 5%, and 1% level, respectively. Bank-level characteristics, institution, and macroeconomic variables are described in the S1 Table.

We find a significant concentration effect for both types of banks. Specifically, the estimated coefficients of our concentration measure (the HH-index) are marginally significant and positive for all three measures of bank efficiency (see Table 5). A similar result is observed for the group of IBs (see Table 6). We may conclude that both banking institutions can achieve higher efficiency if they operate in concentrated markets. Since there is no meaningful difference in the magnitude of the concentration effect between CBs and IBs, we have to reject our hypothesis H3a. However, we observe a significant differential effect of concentration on IBs efficiency when considering the COVID-19 impact. The positive sign of the interaction term of our concentration measure (CR3) and the crisis dummy variable indicates that the concentration effect intensifies during the COVID-19 outbreak (see Table 6). This supports our hypothesis H3b that the concentration effect is more pronounced during the COVID-19 outbreak. However, this effect is significant only in the group of Islamic banking institutions.

Our further analysis indicates that the positive impact of profitability (measured by ROA) is significant only in the group of IBs (see Table 6). This result aligns with [26], who analyzed the efficiency of IBs in the MENA and Asian countries during the period of 2001–2006 and suggested that profitability (among other bank-specific characteristics such as loans, size, and capitalization) is positively correlated with bank efficiency. However, the cost efficiency effect is statistically insignificant, which contradicts the findings of other similar studies (see e.g., [87]). The individual impact of the COVID-19 pandemic on bank efficiency is strongly significant and negative in both samples. Therefore, we may expect that the COVID-19 effects are likely to inflict consolidation in the banking sectors in the MENA region. This finding is confirmed by [29] who report that bank consolidations in many countries in the region “have been proceeding at a rapid pace, leading to a decline in the number of banks and an increase in market concentration” (p. 1). Finally, the positive signs of concentration measures’ coefficients and the negative of its square term signify that there is an inverted U-shaped relationship between concentration and efficiency. Kozak and Wierzbowska [1] state that “the maximum point of the inverted parabola corresponds to the optimal concentration level and maximum bank efficiency, which can be derived from the zero value of the first derivative of the profit efficiency with respect to the concentration” (p. 44). The relationship between the level of banking market concentration and efficiency is shown in S1 Fig.

Are these results informative for bank managers, policymakers, and regulators in the MENA region? We believe they will provide some guidance for bank managers on how to shape their strategies during the COVID-19 pandemic. For example, such strategies should promote bank profitability because we find out that more profitable banks are also more efficient (in the case of IBs). Thus, bank managers should strive to increase operations efficiency of IBs benefiting from the increased level of market concentration in the respective countries. Moreover, regulatory authorities and policymakers should adopt policies that prevent further increases in bank size (in the case of CBs) due to unexpected consolidation in the banking industry caused by the COVID-19 pandemic.

### The ownership structure effect on bank efficiency

The influence of market concentration on bank efficiency might vary depending on the ownership structure. Previous studies report that foreign-owned banks (foreign banks) in developing economies are considered to have better management and technological equipment than government-owned banks (domestic banks), which allows them to achieve better efficiency and a stronger competitive position [88]. Since this relationship is still unexplored for banks in the MENA region, Models 1, 2, and 3, described in Table 4, are estimated for two groups of banks with different ownership structure: government-owned and foreign-owned banks. We present the outcomes of the regression analysis in Table 7 (for HH-index) and Table 8 (for CR3) (see S4 and S5 Files).

**Table 7:** Panel regressions of bank efficiency (All banks, 2006–2020).

	Panel A: Government ownership			Panel B: Foreign ownership		
Efficiency measures	CRS	VRS	SCALE	CRS	VRS	SCALE
	Model 1	Model 2	Model 3	Model 4	Model 5	Model 6
Constant	0.489***	-0.069	-0.043	0.559***	0.747***	0.607***
	(0.000)	(0.500)	(0.657)	(0.000)	(0.000)	(0.000)
*HHI*	0.271*	0.312*	0.331**	0.025	0.230	0.036
	(0.083)	(0.064)	(0.040)	(0.798)	(0.688)	(0.529)
*HHI^2*	-0.238	-0.298	-0.277	-0.001	-0.236	-0.031
	(0.209)	(0.156)	(0.170)	(0.988)	(0.594)	(0.814)
HHI* Islamic	0.467***	0.561***	0.545***	0.197***	0.373**	0.313***
	(0.006)	(0.002)	(0.002)	(0.002)	(0.000)	(0.000)
*ROA*	0.005	0.006	0.007	0.007	0.002	0.003
	(0.412)	(0.337)	(0.266)	(0.883)	(0.670)	(0.547)
C/I * 100	0.047	0.021	0.039	0.003	0.006	0.019
	(0.204)	(0.588)	(0.302)	(0.934)	(0.905)	(0.650)
*Deposit/Assets*	-0.031	-0.065	-0.048	-0.004	-0.012	-0.005
	(0.479)	(0.187)	(0.303)	(0.889)	(0.782)	(0.869)
*Loan/Assets*	0.005	0.093*	0.036*	-0.011	-0.040*	-0.022**
	(0.917)	(0.079)	(0.076)	(0.714)	(0.061)	(0.015)
*Size*	-0.002	-0.007	0.008	0.002	0.006	0.002
	(0.552)	(0.849)	(0.982)	(0.364)	(0.856)	(0.370)
Loan Growth * 100	0.001	0.078	0.032	-0.001	-0.060**	-0.026
	(0.996)	(0.821)	(0.982)	(0.934)	(0.020)	(0.200)
*Liquid Assets*	-0.011	0.101	0.030	0.031	-0.030	0.018
	(0.849)	(0.134)	(0.637)	(0.450)	(0.584)	(0.681)
*Equity/TA*	-0.035	-0.038	-0.032	0.003	-0.024	-0.017
	(0.597)	(0.592)	(0.638)	(0.936)	(0.684)	(0.718)
*GDP Growth*	-0.086	-0.343	-0.053	-0.073	-0.093	-0.093
	(0.670)	(0.146)	(0.813)	(0.560)	(0.605)	(0.514)
*Inflation*	0.331**	0.337*	0.352**	0.037	0.001	0.077
	(0.045)	(0.068)	(0.047)	(0.680)	(0.989)	(0.445)
*Islamic*	-0.111***	-0.131***	-0.112***	-0.059***	-0.114***	-0.068***
	(0.006)	(0.003)	(0.007)	(0.001)	(0.000)	(0.000)
*COVID-19*	-0.066***	-0.063*	-0.044*	-0.059***	-0.038*	-0.032**
	(0.006)	(0.090)	(0.101)	(0.001)	(0.050)	(0.027)
Country Dummy	Yes	Yes	Yes	Yes	Yes	Yes
Year Dummy	Yes	Yes	Yes	Yes	Yes	Yes
Number of Observations	556	556	556	1312	1312	1312
*R*-squared (Overall)	0.5728	0.7594	0.5812	0.4428	0.5736	0.4708

The panel data regressions estimate the relation between bank efficiency and market concentration for the period from 2006 to 2020 while controlling for important bank-level and macroeconomic characteristics. The sample includes 225 banks in 18 countries in the MENA region. Banks included in the sample are either government-owned or foreign-owned banks. The efficiency measures are DEA efficiency scores computed using different approaches (constant returns to scale approach CRS; variable returns to scale approach, VRS; and the ratio of CRS score to VRS score, SCALE). Bank profitability and cost efficiency are proxied with accounting measures: Return on Assets (ROA) and Cost-to-Income (C/I) ratio. The HH-Index is used to measure the level of market concentration. Bank-level characteristics and country-specific variables are computed as of year *t-1*. All the regressions control for year- and country-fixed effects. *, **, and *** indicate statistical significance at the 10%, 5%, and 1% level, respectively. Bank-level characteristics, institution, and macroeconomic variables are described in the S1 Table.

**Table 8:** Panel regressions of bank efficiency (All banks, 2006–2020).

	Panel A: Government-owned			Panel B: Foreign owned		
Efficiency measures	CRS	VRS	SCALE	CRS	VRS	SCALE
	Model 1	Model 2	Model 3	Model 4	Model 5	Model 6
Constant	-0.065	-0.082	-0.061	0.518***	0.733***	0.588***
	(0.507)	(0.439)	(0.554)	(0.000)	(0.000)	(0.000)
*CR3*	0.168	0.198	0.207	0.137	0.102	0.206**
	(0.252)	(0.214)	(0.019)	(0.126)	(0.402)	(0.037)
*CR3^2*	-0.148	-0.213	-0.184	-0.165**	-0.208*	-0.242***
	(0.268)	(0.143)	(0.192)	(0.047)	(0.065)	(0.008)
CR3* Islamic	0.051*	0.131***	0.067**	0.154***	0.319***	0.188***
	(0.108)	(0.000)	(0.046)	(0.000)	(0.000)	(0.000)
*ROA*	0.005	0.005	0.007	0.006	0.003	0.003
	(0.362)	(0.416)	(0.260)	(0.899)	(0.624)	(0.480)
C/I * 10	0.056	0.026	0.042	0.0002	0.0002	0.0002
	(0.120)	(0.497)	(0.267)	(0.589)	(0.704)	(0.511)
*Deposit/Assets*	-0.031	-0.044	-0.032	0.002	0.028	0.002
	(0.487)	(0.361)	(0.490)	(0.931)	(0.499)	(0.943)
*Loan/Assets*	0.031	0.081	0.027	-0.029	-0.060	-0.035
	(0.522)	(0.124)	(0.592)	(0.344)	(0.156)	(0.302)
Size*10	-0.002	-0.003	-0.001	-0.0008	-0.003	0.002
	(0.434)	(0.392)	(0.666)	(0.972)	(0.326)	(0.934)
*Loan Growth*	0.0005	-0.0007	0.0003	0.012	0.006	0.016
	(0.860)	(0.818)	(0.906)	(0.499)	(0.814)	(0.439)
*Liquid Assets*	0.058	0.112	0.037	0.030	0.021	0.026
	(0.346)	(0.096)	(0.562)	(0.442)	(0.693)	(0.554)
*Equity/TA*	-0.018	-0.045	-0.033	0.005	-0.018	-0.009
	(0.783)	(0.530)	(0.626)	(0.895)	(0.754)	(0.843)
*GDP Growth*	-0.036	-0.288	-0.010	0.024	-0.023	-0.053
	(0.868)	(0.225)	(0.965)	(0.848)	(0.895)	(0.706)
*Inflation*	0.196**	0.261*	0.289*	0.009	-0.098	0.005
	(0.038)	(0.101)	(0.100)	(0.913)	(0.423)	(0.953)
*Islamic*	-0.030	-0.042	-0.018	-0.054***	-0.092***	-0.044**
	(0.228)	(0.120)	(0.494)	(0.001)	(0.000)	(0.011)
*COVID-19*	-0.007	-0.012	-0.002	-0.054**	-0.123***	-0.065**
	(0.984)	(0.761)	(0.960)	(0.037)	(0.000)	(0.022)
Country Dummy	Yes	Yes	Yes	Yes	Yes	Yes
Year Dummy	Yes	Yes	Yes	Yes	Yes	Yes
Number of Observations	556	556	556	1312	1312	1312
*R*-squared (Overall)	0.5549	0.7664	0.5829	0.4679	0.6033	0.5043

The panel data regressions estimate the relation between bank efficiency and market concentration for the period from 2006 to 2020 while controlling for important bank-level and macroeconomic characteristics. The sample includes 225 banks in 18 countries in the MENA region. Banks included in the sample are either government-owned or foreign-owned banks. The efficiency measures are DEA efficiency scores computed using different approaches (constant returns to scale approach CRS; variable returns to scale approach, VRS; and the ratio of CRS score to VRS score, SCALE). Bank profitability and cost efficiency are proxied with accounting measures: Return on Assets (ROA) and Cost-to-Income (C/I) ratio. The concentration ratio (CR3) is used to measure the level of market concentration. Bank-level characteristics and country-specific variables are computed as of year *t-1*. All the regressions control for year- and country-fixed effects. *, **, and *** indicate statistical significance at the 10%, 5%, and 1% level, respectively. Bank-level characteristics, institution, and macroeconomic variables are described in S1 Table.

The results in Table 7 indicate that the banking market concentration influences the efficiency of government and foreign banks in a significantly different way. Specifically, the market concentration effect is strongly significant and positive for the government-owned bank (Panel A) but insignificant in the group of foreign-owned banks (Panel B). Our results are robust to using different alternative measures of bank efficiency; the estimated coefficients for the concentration measure (the HH-index in this case) have the same signs and similar values across all three efficiency scores (CRS, VRS, and SCALE). The significant difference in the response of the two types of banks (government vs. foreign) to changes in the concentration level of the banking market supports our last hypothesis (H4). One possible reason for the observed difference might be the fact that government-owned banks in the MENA countries may have informational advantages relative to foreign banks and, thus, better efficiency. Furthermore, the concentration effect is more pronounced for government Islamic banking institutions. This result somewhat supports [24] in their study of Malaysian banks that “local Islamic banks scored higher technical efficiency and scale efficiency, but foreign Islamic banks scored higher pure technical efficiency” (p. 1). Sufian and Kamarudin [89] examine the revenue efficiency of Islamic banks operating in Southeast Asian countries and find that revenue efficiency of domestic Islamic banks is higher compared to foreign Islamic banks. Similar results are reported by Kamarudin et al. [90].

We do not find evidence that more profitable banks (either government-owned or foreign-owned) are more efficient. Moreover, bank-specific characteristics have only a marginal effect on efficiency. As expected, the COVID-19 effect is negative and strongly significant. That is, the efficiency level of both government-owned and foreign-owned banks decreased during the COVID-19 outbreak. The results from additional tests using CR3 as an alternative concentration measure are similar to those reported in Table 8. However, the market concentration effect is no longer significant for government-owned banks and shows a non-linear association with efficiency only in the group of foreign-owned banks. Our findings reported in Table 8 lend further support to our last hypothesis (H4), according to which market concentration effect on bank efficiency should be significantly different between government- and foreign-owned banks in the MENA region. The policy implication of this finding is that decision-makers in the MENA countries may consider introducing new rules and regulations to support government IBs’ effectively. At the same time, bank managers should focus more on improving the operations efficiency of IBs benefiting from the increased level of market concentration in the respective countries.

### Robustness checks and alternative specifications

In this section, we conduct a few robustness checks of our main findings. First, we follow Mirzae et al. [48] and re-estimate Eq (1) using a single efficiency score that is calculated as the average across the three efficiency measures (CRS, VRS, and SCLAE). The results (not reported here to conserve space) indicate that the concentration effect on bank efficiency remains robust, and this effect is more pronounced when the banking institution is Islamic. The COVID-19 pandemic effect is negative and statistically significant as before. Second, we split the sample into two sub-samples (CBs and IBs) and employ the regression models in Tables 5 and 6 using the average efficiency score (AVERAGE). The results do not change significantly. These findings strengthen the conclusion that banking market concentration indeed matters for bank efficiency in the MENA region.

Next, we supplement our analysis with few alternative tests. First, since the empirical models in this paper are dynamic panel models, the ordinary least squares estimator (OLS) and fixed effect estimators are biased. To solve this problem, [91] proposed the generalized method of moments (GMM) estimators, which include difference GMM estimators and system GMM estimators. According to [92, 93], system GMM estimator augments the difference GMM estimator by estimating simultaneously in differences and levels, which makes up for the deficiency of the difference GMM estimator and enhances the validity of instruments in the difference equation. The system GMM estimators include one-step and two-step estimators. In this study, we use a two-step system GMM estimator developed by [92], with Windmeijer [94] corrected standard errors. System GMM is implemented by command xtabond2 on the STATA package. The bank-level explanatory variables are considered predetermined, while macroeconomic and financial variables are treated as strictly exogenous variables. Because Roodman [95] suggests keeping the number of instruments smaller than a number of groups, we use only the first and second lag of the predetermined variables as instruments. The results for GMM tests of the effect of market concentration on efficiency are reported in Table 9 using the same variables as in Table 4 (S1 File). We also run the Hansen’s J test of over-identification and the Sargan test results, which confirm the validity of the instruments. Again, we observe a strong positive influence of the market concentration on bank efficiency. The results also confirm our expectations for the nonlinear association between market concentration and bank efficiency.

**Table 9:** Panel regressions (GMM estimator) of bank efficiency (All Banks, 2006–2020).

	Panel A: HH-INDEX			Panel B: CR3		
Efficiency measures	CRS	VRS	SCALE	CRS	VRS	SCALE
	Model 1	Model 2	Model 3	Model 4	Model 5	Model 6
Constant	1.998	2.982	2.463	0.266	0.578	0.047
	(0.262)	(0.178)	(0.157)	(0.701)	(0.795)	(0.949)
*HH-INDEX*	-0.713***	-0.230	-0.763***			
	(0.005)	(0.672)	(0.007)			
*HH-INDEX^2*	0.772**	0.310	0.831**			
	(0.014)	(0.609)	(0.013)			
*CR3*				0.395*	0.787*	0.349*
				(0.097)	(0.103)	(0.101)
*CR3^2*				-0.350*	-0.749*	-0.312*
				(0.058)	(0.067)	(0.080)
HH-INDEX* *Islamic*	0.511**	0.865**	0.530**			
	(0.040)	(0.043)	(0.043)			
CR3* *Islamic*				0.044	0.668*	0.041
				(0.793)	(0.060)	(0.803)
*ROA*	-0.002	-0.008	-0.002	-0.003	-0.005	-0.003
	(0.713)	(0.242)	(0.650)	(0.603)	(0.444)	(0.607)
*C/I* * *100*	0.022	0.007	0.042	0.482	0.037	0.371
	(0.578)	(0.533)	(0.371)	(0.589)	(0.496)	(0.728)
*Deposit/Assets*	-0.056	-0.030	-0.072*	0.208	0.011	0.197
	(0.169)	(0.600)	(0.076)	(0.152)	(0.865)	(0.233)
*Loan/Assets*	0.045	0.010	0.049	-0.089	-0.026	-0.114
	(0.464)	(0.882)	(0.413)	(0.661)	(0.750)	(0.598)
*Size*	0.003	0.006	0.005	0.005	0.005	0.010
	(0.329)	(0.337)	(0.146)	(0.393)	(0.334)	(0.189)
*Loan Growth*	0.002	0.003	0.001	0.010	0.001	0.019
	(0.698)	(0.692)	(0.901)	(0.553)	(0.984)	(0.478)
*Liquid Assets*	-0.008	-0.009	-0.005	-0.604***	-0.142***	-0.675***
	(0.883)	(0.926)	(0.992)	(0.001)	(0.002)	(0.001)
*Equity/TA*	-0.006	0.076	-0.021	0.063	0.026	0.018
	(0.911)	(0.400)	(0.689)	(0.765)	(0.792)	(0.941)
*GDP Growth*	-0.572	-0.333	-0.368	0.510	0.338	0.618
	(0.206)	(0.694)	(0.603)	(0.247)	(0.742)	(0.176)
*Inflation*	-0.452	-0.903	-0.743	-0.331	-0.106	-0.217
	(0.296)	(0.174)	0.153)	(0.202)	(0.878)	(0.437)
*Lag_1(Efficiency)*	-0.020	-0.046	-0.022	-0.041	-0.032	-0.042
	(0.641)	(0.720)	(0.957)	(0.260)	(0.452)	(0.224)
*Islamic*	0.114	0.134	0.027	0.108*	0.117*	0.108*
	(0.992)	(0.423)	(0.837)	(0.062)	(0.054)	(0.081)
*COVID-19*	-0.180***	-0.214***	-0.163***	-0.152***	-0.176***	-0.142***
	(0.000)	(0.000)	(0.000)	(0.000)	(0.002)	(0.000)
Country Dummy	Yes	Yes	Yes	Yes	Yes	Yes
Number of observations	2899	2899	2899	2899	2899	2899
AR (1) test (*p*-value)	0.000	0.000	0.000	0.000	0.000	0.000
AR (2) test (*p*-value)	0.092	0.978	0.135	0.094	0.115	0.086
Hansen’s J test of over-identification (*p-value*)	0.570	0.341	0.614	0.669	0.581	0.513
Sargan test of overidentifying restrictions (*p-value*)	0.764	0.648	0.550	0.748	0.677	0.514

The panel data regressions estimate the relation between bank efficiency and market concertation using the GMM estimator for the period of from 2006 to 2020 while controlling for important bank-level and macroeconomic characteristics. The sample includes 225 banks in 18 countries in the MENA region. Banks included in the sample are either conventional banks or Islamic banks. The efficiency measures are DEA efficiency scores computed using different approaches (constant returns to scale approach CRS; variable returns to scale approach, VRS; and the ratio of CRS score to VRS score, SCALE). Bank profitability and cost efficiency are proxied with accounting measures: Return on Assets (ROA) and Cost-to-Income (C/I) ratio. The HH-Index and the concentration ratio (CR3) are used to measure the level of market concentration. Bank-level characteristics and country-specific variables are computed as of year *t-1*. All the regressions control for year- and country-fixed effects. *, **, and *** indicate statistical significance at the 10%, 5%, and 1% level, respectively. Bank-level characteristics, institution, and macroeconomic variables are described in the S1 Table.

Second, for robustness check purposes, we employ two alternative measures of bank profitability–return on equity (ROE) and net interest margin (NIM). The results for ROE reported in S4 Table are insignificant; however, the NIM variable shows a positive and significant association with efficiency, which confirms the findings of other similar studies on MENA bank efficiency (see e.g., [23]). Finally, instead of using CR3, we run our baseline model (1) with an alternative measure of market concentration computed as the share of top five banks in total assets of a country’s banking sector (CR5). The results for CR5 are reported in S5 Table and indicate that the concentration ratio is significantly positively associated with different measures of bank efficiency. These results confirm our findings based on the HHI indicator (see Models 1, 2 and 3). When the analysis is contended separately for each type of banks (IBs and CBs), the outcomes of the regressions indicate that the relationship between concentration and efficiency is strongly significant in the group of CBs but irrelevant for IBs (the results are available on request).

Are these results relevant for MENA banks? Previous studies report that increased efficiency positively influences banks’ financial stability and credit risk reduction (see [96, 97]). Their findings suggest that banks with better efficiency are more profitable but less stable in competitive environments. We complement these studies by exploring the trade-off between concentration and efficiency and its impact on bank stability in the MENA region. Based on our results, we suggest that banks can benefit of increased efficiency and more competitive markets in order to enhance their profitability and financial stability only if appropriate income diversification strategies are implemented in a sustained way. Moreover, though bank managers are often driven by short-term results, the adoption of appropriate risk management strategies that increase bank stability will allow, in the long term, to mitigate the institutional risk imposed by the various regulatory reform initiatives. These results are particularly relevant in the context of current pandemic crisis (COVID-19), characterized by an increase in credit risk and operating costs.

## Conclusions

In this paper, we analyze the impact of market concentration on bank efficiency in the MENA region, incorporating the effect of the COVID-19 outbreak. We report a number of interesting results that have strong policy implications.

First, our analysis reveals a positive association between market concentration and bank efficiency. The concentration thus creates favorable conditions for banks to increase their profit efficiency. However, the increased competition between concentrated banks can increase risk taking and decrease efficiency, which will harm the banking system’s financial stability. Our results remain robust to different alternative measures of concentration (the HH-index and CR3). Our findings align with the studies on bank efficiency in other developing countries (see e.g., [1] for the SECE region). Specifically, we observe a nonlinear (inverted U-shaped) relationship between market concentration and bank efficiency. This means that, in the case of low and highly concentrated markets, an additional increase in concentration will provide less incentives for improvement in bank efficiency, with the opposite effect for moderately concentrated markets. Because banking markets in the MENA countries are characterized as moderately concentrated [84], this provides room for further improvements in bank efficiency, specifically in the Islamic banking market. However, regulatory authorities concerned with improving financial stability in the MENA region should proceed carefully since policies that amplify further consolidation in the banking sector may reduce competition and create oligopolistic market structures.

Second, we contribute to the academic debate on the efficiency of different bank systems (conventional and Islamic) that co-exist in MENA and other developing regions. The empirical literature provides mixed evidence depending on the context: studies on individual countries report that IBs are more efficient than CBs and vice versa, whereas papers reporting cross-country evidence find no difference between the two types of banks. Our analysis reveals a strong differential effect for IBs. Specifically, market concentration effect on bank efficiency is more pronounced when the banking institution is Islamic. To test this notion, we split the sample into two groups (CBs and IBs) and repeat the baseline analysis separately for each group of banks. We find a similar (positive) impact of market concentration on the efficiency of either type of bank. However, the concentration effect is more pronounced during the COVID-19 outbreak only in the group of IBs. The positive sign of interaction term of the concentration measure (CR3 in this case) and the crisis dummy variable indicates that any increase in the concentration level during the COVID-19 outbreak intensifies IBs efficiency. As IBs show higher efficiency levels and stability than their conventional counterparts during the pandemic crisis (see S3 Table), Islamic banks “can be entrusted with a more decisive and important role in post-COVID-19 revival” [97, p. 2].

Finally, there is limited empirical evidence of whether the relationship between market concentration and efficiency depends on the banks’ ownership structure. We contribute to this area by addressing the question of whether the concentration effect is similar for banks that are government-owned or have foreign ownership. Our analysis indicates that market concentration effect is strongly significant and positive for the government-owned bank but insignificant in the group of foreign-owned banks. One possible reason for this difference might be that foreign banks in the MENA countries suffer more from bad institutional framework in the host country than government-owned (domestic) ones. Moreover, the concentration effect is more pronounced for domestic Islamic banking institutions. This result somewhat supports the finding of [24] for Malaysian banks that the technical efficiency and scale efficiency of domestic IBs are higher. In comparison, foreign IBs operate at higher pure technical efficiency.

Our findings provide strong implications for bank managers, policymakers, and regulators in the MENA region. Specifically, the results related to IBs efficiency can guide bank managers on how to shape their strategies during the COVID-19 pandemic. Though our findings relate to the COVID-19 outbreak only, they provide guidance to the managers to enhance bank profitability because it is positively associated with bank efficiency level. Moreover, though bank managers are often driven by short-term results, the adoption of appropriate risk management strategy during the crisis times (such as COVID-19) that increase bank stability will allow, in the long term, to mitigate the institutional risk imposed by the various regulatory reform initiatives. An example of such initiatives are policies oriented to increase the level of concentration to improve the financial stability of the banking sector. However, policy makers and regulators should be cautious in implementing policies that increase market concentration without considering the potential negative effects on competition, consumers, and systemic risk. Therefore, regulatory authorities and policy makers concerned with improving financial stability in the MENA region should proceed differently depending on the level of concentration and the type of banking system. For example, they may adopt policies that prevent the further increase in banking size (in the case of CBs) through additional consolidation in the banking industry owing to the COVID-19 pandemic. Referring to IBs, as they show better efficiency and stability than their conventional counterparts during the recent crisis, increased concentration will most likely positively affect the efficiency of Islamic banks’ operations; therefore, these institutions can be entrusted with a more decisive and important role in the post-COVID-19 revival.

However, the main limitation of our analysis is related to the fact that the effect of the global pandemic crisis on bank efficiency is analyzed using a limited period of one year (the last year of the sample period that coincides with the COVID-19 outbreak). Therefore, further investigation of the COVID-19 effect on bank efficiency and profitability is required using an extended period of global crisis impact. This will be the area of our future research.

## Supporting information

S1 Table List of dependent and explanatory variables. (DOCX)

S2 Table Summary statistics for input and output variables used in the DEA analysis [48, 78, 98–100]. (DOCX)

S3 Table Constant returns-to-scale’s (CRS) efficiency scores by country, year, and bank type (2006–2020). (DOCX)

S4 Table Panel regressions with different profitability measures (ROE and NIM). (DOCX)

S5 Table Panel regressions with alternative concentration measure (CR5). (DOCX)

S1 Fig U-shaped relationship between concentration and efficiency. (DOCX)

S1 Data Primary data sources used to compute the variables and the sample statistics (Tables 1 and 2). (XLSX)

S1 File Primary data sources used in the regression analysis run in STATA 11 package (Total sample). (XLSX)

S2 File Primary data sources used in the regression analysis run in STATA 11 package (Conventional banks sample). (XLSX)

S3 File Primary data sources used in the regression analysis run in STATA 11 package (Islamic banks sample). (XLSX)

S4 File Primary data sources used in the regression analysis run in STATA 11 package (Government banks sample). (XLSX)

S5 File Primary data sources used in the regression analysis run in STATA 11 package (Foreign banks sample). (XLSX)
