# Correction: Efficiency, market concentration and bank performance during the COVID-19 outbreak: Evidence from the MENA region

**DOI:** 10.1371/journal.pone.0315310

**Published:** 2024-12-05

**Authors:** Miroslav Mateev, Muhammad Usman Tariq, Ahmad Sahyouni

The affiliation for the second author is incorrect. The correct affiliation is not indicated. Muhammad Usman Tariq is not affiliated with #1 but with: Abu Dhabi University, Abu Dhabi, UAE.
