# Supplementary material for: Efficiency, market concentration and bank performance during the COVID-19 outbreak: Evidence from the MENA region
Source: PLoS One. 2023 May 10;18(5):e0285403. doi: 10.1371/journal.pone.0285403 (PMC10171612; doi:10.1371/journal.pone.0285403)
Supplement: S1 Table — (DOCX) [file pone.0285403.s001.docx]

**S1 Table**

**Dependent and explanatory variables**

| **Variable** | **Explanation** | **Data Source** |
| --- | --- | --- |
| **Dependent Variables** |  |  |
| Return on Assets (ROA) | Net profit to the average total assets of a bank | Authors’ calculations based on Bankscope/Orbis |
| Return on Assets (ROE) | Net profit to the average total equity of a bank | Authors’ calculations based on Bankscope/Orbis |
| Net interest margin (NIM) | Bank interest income minus bank interest expenses as a percentage of earning assets | Authors’ calculations based on Bankscope/Orbis |
| ***Efficiency measures*** |  |  |
| *DEA 1 (CRS)* | Bank efficiency score computed using constant returns to scale approach. | Authors’ calculations based on Bankscope/Orbis |
| *DEA 2 (VRS)* | Bank efficiency score computed using variable returns to scale approach. | Authors’ calculations based on Bankscope/Orbis |
| *DEA 3 (SCALE)* | Bank efficiency score computed as the ratio of CRS score/VRS score. | Authors’ calculations based on Bankscope/Orbis |
| ***Market Competition*** |  |  |
| HH-index | Herfindahl-Hirschman index (HHI) is a measure of market concentration calculated as the sum of the squared market shares for each  bank in a country. | Authors’ calculations based on Bankscope/ Orbis |
| Concentration ratio (CR3) | The share of top three banks in total assets of a country’s banking sector | Authors’ calculations based on Bankscope/ Orbis |
| ***Bank-level Characteristics*** |  |  |
| Deposit/Assets | Deposits divided by total assets | Authors’ calculations based on Bankscope/ Orbis |
| Loan/Assets | Loans divided by total assets | Authors’ calculations based on Bankscope/ Orbis |
| Size | Natural logarithm of a bank’s total assets | Authors’ calculations based on Bankscope/ Orbis |
| Loan Growth | Rate of growth of gross loans | Authors’ calculations based on Bankscope/ Orbis |
| Liquid Assets | Liquid assets divided by total assets | Orbis Bank Focus and annual reports (2006-2020) |
| Equity/Total Assets | The ratio of equity to total assets of a bank | Orbis Bank Focus and annual reports (2006-20201) |
| ***Risk measures*** |  |  |
| Non-performing loans/ Gross loans (NPL/GL) | Bank non-performing loans divided by gross loans | Authors’ calculations based on Bankscope/ Orbis |
| Distance to default (Z-score) | Z-score is the distance to default calculated the abank's ROA plus the capital-to-asset ratio divided by the standard deviation of ROA over the period of three years | Authors’ calculations based on Bankscope/ Orbis |
| ***Ownership Indicators*** |  |  |
| Ownership concentration | The level of ultimate ownership held by the largest shareholder | Bankscope/Orbis and annual reports (2006-2020) |
| Government ownership | Proportion of equity held by the government | Bankscope/Orbis and annual reports (2006-2020) |
| Foreign ownership | Proportion of equity held by the foreign shareholders | Bankscope/Orbis and annual reports (2006-2020) |
| ***Quality of institutions*** |  |  |
| Institution | The index of institution is the average of six indicators: 1) voice and accountability, (2) political stability and absence of violence, (3) government effectiveness, (4) regulatory quality, (5) rule of law, and (6) control of corruption. | The World Bank, World Development Indicators 2006-2020 |
| ***Macroeconomic Variables*** |  |  |
| GDP Growth | Annual percentage growth rate of GDP (constant 2005 U.S. dollars), proxy for market size | The World Bank Development Indicators, 2006-2020 |
| Inflation | Inflation is measured by the consumer price index, in percent | The World Bank Development Indicators, 2006-2020 |
