# Supplementary material for: Efficiency, market concentration and bank performance during the COVID-19 outbreak: Evidence from the MENA region
Source: PLoS One. 2023 May 10;18(5):e0285403. doi: 10.1371/journal.pone.0285403 (PMC10171612; doi:10.1371/journal.pone.0285403)
Supplement: S2 Table — (DOCX) [file pone.0285403.s002.docx]

**S2 Table**

**Summary statistics for input and output variables used in the DEA analysis.**

DEA can be either input- or output-orientated. Under constant returns to scale (CRS) both input- and output- oriented DEA models produce the same efficiency scores, while under variable returns to scale (VRS), the efficiency scores in two models may be different. The reason is that input-oriented DEA method defines the frontier by seeking the maximum possible proportional reduction in input usage, with output levels held constant, for each DMU [48]. While, for the output-orientated case, the DEA method seeks the maximum proportional increase in output production, with input levels held fixed. Empirically, we follow the intermediation approach to select inputs and outputs [98, 99, 100]. According to this approach, banks are viewed as financial intermediaries that borrow funds from surplus economic units and use labor and capital to transform such funds to earning assets (loans and other-earning assets) and non-interest income.

Guided by [48, 78], we use the following variables as inputs: (i) staff costs as a proxy for labor inputs, (ii) fixed assets as a proxy for capital input, (iii) total deposits as a proxy for financial input, and (iv) impaired loans to account for the credit risk. Accordingly, we consider three outputs: (i) gross customer loans; (ii) other earning assets, including investment securities, loans and advances to banks, and other investment; and (iii) non-interest income, including net fees and commissions, net gains/losses on trading and derivatives, and other operating income. Mirzae et al. [48] claim that non-interest income can be viewed as a proxy for off-balance-sheet activities, which is an important component of banking business.

Table 2A in the main text presents the summary statistics of DEA’s input and output variables of each group of banks (CBs and IBs). The summary statistics of input variables for our IBs sample show that, on average, IBs have 0.6% staff costs, which is less than those reported for the CBs sample at 1.3%. This suggests that IBs are, to some extent, superior to CBs in managing overheads. However, the difference in means is statistically insignificant when comparing the staff costs between the two groups. On the other hand, IBs have a similar ratio of fixed assets, compared to CBs, which is 2.7% vs. 3.0%. The mean difference is statistically insignificant when comparing the two samples. This implies that CBs and IBs have a similar level of financial leverage. Concerning the deposit ratio, it is higher in the group of IBs, compared to their CBs counterparts (69% vs. 56%); the mean difference is highly statistically significant at 1% significance. Finally, for bank credit risk, we surprisingly find that IBs have a higher level of impaired loans to total asset ratio, and this difference is strongly significant when comparing the two groups of banks IBs. This indicates that a typical IB is riskier (at least in terms of loan portfolio quality) than a typical conventional bank. This result aligns with [48] findings. With regard to output variables, we find the mean of loan ratio is higher for IBs sample than those reported for CBs sample (47.8% vs. 41.3%), and the differences are statistically significant at 5% significance. The ratio of other earning assets to total assets is rather low for IBs, compared to those for CBs (24.9% vs. 36.4%), and the difference is strongly significant when comparing the two samples. This suggests that IBs are relatively more active in holding earning assets than loans. Finally, IBs earn slightly less income from non-traditional bank activities than CBs (0.6% vs. 1.4%); the mean difference is highly statistically significant.
