# Supplementary material for: Efficiency, market concentration and bank performance during the COVID-19 outbreak: Evidence from the MENA region
Source: PLoS One. 2023 May 10;18(5):e0285403. doi: 10.1371/journal.pone.0285403 (PMC10171612; doi:10.1371/journal.pone.0285403)
Supplement: S3 Table — (DOCX) [file pone.0285403.s003.docx]

**S3 Table**

**This table presents the constant returns-to-scale’s (CRS) efficiency scores** **by country, year, and bank type (2006-2020).**

| **Country/Year** | **2006** | **2007** | **2008** | **2009** | **2010** | **2011** | **2012** | **2013** | **2014** | **2015** | **2016** | **2017** | **2018** | **2019** | **2020** | **Mean** |
| --- | --- | --- | --- | --- | --- | --- | --- | --- | --- | --- | --- | --- | --- | --- | --- | --- |
| United Arab Emirates | 0.74 | 0.61 | 0.84 | 0.88 | 0.61 | 0.41 | 0.57 | 0.48 | 0.45 | 0.50 | 0.57 | 0.56 | 0.65 | 0.53 | 0.36 | 0.58 |
| Bahrain | 0.75 | 0.36 | 0.66 | 0.81 | 0.38 | 0.55 | 0.62 | 0.60 | 0.47 | 0.50 | 0.54 | 0.50 | 0.66 | 0.55 | 0.27 | 0.55 |
| Algeria | n.a. | n.a. | n.a. | n.a. | n.a. | 0.62 | 0.71 | 0.69 | 0.61 | 0.66 | 0.60 | 0.55 | n.a. | n.a. | 0.01 | 0.56 |
| Egypt | 0.87 | 0.49 | 0.91 | 0.86 | 0.50 | 0.30 | 0.56 | 0.51 | 0.51 | 0.49 | 0.18 | 0.36 | 0.66 | 0.44 | 0.16 | 0.52 |
| Israel | 0.59 | 0.54 | 0.79 | 0.76 | 0.58 | 0.50 | 0.71 | 0.64 | 0.58 | 0.58 | 0.09 | 0.19 | 0.40 | 0.46 | 0.09 | 0.50 |
| Iraq | n.a. | n.a. | n.a. | n.a. | n.a. | 0.54 | 0.62 | 0.53 | 0.51 | 0.67 | 0.16 | 0.30 | 0.24 | 0.49 | 0.13 | 0.42 |
| Iran | n.a. | 0.45 | 0.70 | 0.88 | 0.74 | 0.70 | 0.69 | 0.60 | 0.56 | 0.48 | 0.42 | 0.29 | n.a. | n.a. | 0.12 | 0.55 |
| Jordan | 0.54 | 0.40 | 0.58 | 0.58 | 0.28 | 0.20 | 0.76 | 0.30 | 0.34 | 0.38 | 0.47 | 0.40 | 0.32 | 0.41 | 0.06 | 0.40 |
| Kuwait | 0.79 | 0.44 | 0.73 | 0.66 | 0.55 | 0.37 | 0.52 | 0.45 | 0.51 | 0.60 | 0.63 | 0.50 | 0.34 | 0.49 | 0.19 | 0.52 |
| Lebanon | 0.47 | 0.36 | 0.59 | 0.78 | 0.60 | 0.75 | 0.52 | 0.30 | 0.38 | 0.42 | 0.30 | 0.26 | 0.06 | 0.39 | 0.05 | 0.42 |
| Morocco | n.a. | n.a. | 0.95 | 1.00 | 0.91 | 0.80 | 0.64 | 0.80 | 0.80 | 0.75 | 0.79 | 0.75 | 0.67 | 0.84 | 0.18 | 0.76 |
| Oman | 0.71 | 0.55 | 0.77 | 0.77 | 0.43 | 0.53 | 0.38 | 0.30 | 0.37 | 0.43 | 0.36 | 0.55 | 0.50 | 0.42 | 0.70 | 0.52 |
| Palestinian Territory | n.a. | n.a. | n.a. | n.a. | n.a. | 0.57 | 0.45 | 0.28 | 0.34 | 0.42 | 0.38 | 0.47 | 0.60 | 0.44 | 0.52 | 0.45 |
| Qatar | 0.76 | 0.72 | 0.91 | 0.90 | 0.79 | 0.77 | 0.73 | 0.37 | 0.42 | 0.48 | 0.65 | 0.53 | 0.25 | 0.52 | 0.81 | 0.64 |
| Saudi Arabia | 0.87 | 0.56 | 0.77 | 0.73 | 0.62 | 0.72 | 0.75 | 0.55 | 0.63 | 0.77 | 0.69 | 0.62 | 0.67 | 0.68 | 0.67 | 0.69 |
| Syrian Arab Republic | n.a. | n.a. | n.a. | n.a. | n.a. | 0.63 | 0.60 | 0.69 | 0.57 | 0.35 | 0.44 | 0.34 | 0.79 | 0.54 | 0.21 | 0.52 |
| Tunisia | n.a. | n.a. | n.a. | n.a. | n.a. | 0.64 | 0.70 | 0.69 | 0.60 | 0.49 | 0.55 | 0.53 | 0.76 | 0.63 | 0.54 | 0.61 |
| Yemen | n.a. | n.a. | n.a. | n.a. | n.a. | 0.66 | 0.62 | 0.49 | 0.47 | 0.32 | 0.33 | 0.29 | n.a. | n.a. | 0.06 | 0.40 |
|  |  |  |  |  |  |  |  |  |  |  |  |  |  |  |  |  |
| **Bank Type** |  |  |  |  |  |  |  |  |  |  |  |  |  |  |  |  |
| Conventional | 0.74 | 0.52 | 0.80 | 0.81 | 0.59 | 0.53 | 0.62 | 0.50 | 0.50 | 0.50 | 0.42 | 0.43 | 0.54 | 0.51 | 0.27 | 0.55 |
| Islamic | 0.73 | 0.55 | 0.69 | 0.73 | 0.60 | 0.52 | 0.59 | 0.51 | 0.48 | 0.51 | 0.50 | 0.46 | 0.54 | 0.52 | 0.35 | 0.56 |
