# Supplementary material for: Efficiency, market concentration and bank performance during the COVID-19 outbreak: Evidence from the MENA region
Source: PLoS One. 2023 May 10;18(5):e0285403. doi: 10.1371/journal.pone.0285403 (PMC10171612; doi:10.1371/journal.pone.0285403)
Supplement: S5 Table — (DOCX) [file pone.0285403.s005.docx]

**S4-2 Table**

**Panel regressions with alternative concentration measure (CR5)**

|  | **Panel A: HHI** | | | **Panel B: CR5** | | |
| --- | --- | --- | --- | --- | --- | --- |
| **Efficiency measures** | **CRS** | **VRS** | **SCALE** | **CRS** | **VRS** | **SCALE** |
|  | **Model 1** | **Model 2** | **Model 3** | **Model 4** | **Model 5** | **Model 6** |
| Constant | 0.387*** | 0.630*** | 0.449*** | -0.108 | -0.038 | -0.062 |
|  | (0.000) | (0.0001) | (0.000) | (0.245) | (0.731) | (0.516) |
| *HHI* | 0.134** | 0.157* | 0.124* |  |  |  |
|  | (0.036) | (0.064) | (0.071) |  |  |  |
| *HHI^2* | -0.136* | -0.187* | -0.134* |  |  |  |
|  | (0.087) | (0.076) | (0.101) |  |  |  |
| *CR5* |  |  |  | 1.631*** | 2.034*** | 1.425*** |
|  |  |  |  | (0.000) | (0.000) | (0.000) |
| *CR5^2* |  |  |  | -1.427*** | -1.751*** | -1.208*** |
|  |  |  |  | (0.000) | (0.000) | (0.000) |
| *HHI* Islamic* | 0.499*** | 0.846*** | 0.593*** |  |  |  |
|  | (0.000) | (0.000) | (0.000) |  |  |  |
| *CR5* Islamic* |  |  |  | -0.044 | -0.067 | -0.040 |
|  |  |  |  | (0.311) | (0.200) | (0.373) |
| *ROA* | 0.002 | 0.007 | 0.003 | 0.005** | 0.002* | 0.006*** |
|  | (0.370) | (0.824) | (0.015) | (0.043) | (0.093) | (0.009) |
| *C/I * 100* | 0.004 | 0.004 | 0.011 | 0.003 | 0.041 | 0.021 |
|  | (0.924) | (0.939) | (0.817) | (0.947) | (0.531) | (0.715) |
| *Deposit/Assets* | -0.037* | -0.041* | -0.038* | 0.009 | -0.011 | 0.014 |
|  | (0.065) | (0.101) | (0.079) | (0.754) | (0.747) | (0.624) |
| *Loan/Assets* | 0.019 | 0.023 | 0.017 | 0.016 | 0.049 | -0.006 |
|  | (0.363) | (0.427) | (0.456) | (0.633) | (0.234) | (0.857) |
| *Size * 10* | 0.004 | 0.003 | 0.005 | -0.005 | 0.004 | 0.003 |
|  | (0.757) | (0.879) | (0.768) | (0.784) | (0.860) | (0.862) |
| *Loan Growth* | -0.001 | -0.002 | -0.002 | 0.005 | 0.005 | 0.006 |
|  | (0.677) | (0.507) | (0.522) | (0.220) | (0.329) | (0.131) |
| *Liquid Assets* | 0.020 | 0.002 | 0.021 | 0.023 | 0.029 | 0.034 |
|  | (0.433) | (0.499) | (0.438) | (0.505) | (0.480) | (0.342) |
| *Equity/TA* | 0.006 | 0.007 | 0.001 | 0.020 | 0.059 | 0.026 |
|  | (0.820) | (0.401) | (0.958) | (0.565) | (0.170) | (0.480) |
| *GDP Growth* | 0.063 | 0.109 | 0.055 | -0.022 | 0.099 | -0.126 |
|  | (0.475) | (0.349) | (0.559) | (0.827) | (0.344) | (0.268) |
| *Inflation* | 0.032 | 0.007 | 0.025 | -0.099* | -0.259*** | -0.091** |
|  | (0.597) | (0.929) | (0.693) | (0.079) | (0.000) | (0.028) |
| *Islamic* | -0.106*** | -0.171*** | -0.106*** | -0.037 | -0.013 | -0.032 |
|  | (0.000) | (0.000) | (0.000) | (0.264) | (0.739) | (0.346) |
| *COVID-19* | -0.008 | -0.002 | -0.006 | -0.073*** | -0.001* | -0.107** |
|  | (0.613) | (0.927) | (0.708) | (0.000) | (0.070) | (0.044) |
| Country Dummy | Yes | Yes | Yes | Yes | Yes | Yes |
| Year Dummy | Yes | Yes | Yes | Yes | Yes | Yes |
| Number of Observations | 3347 | 3347 | 3347 | 3347 | 3347 | 3347 |
| *R*-squared (Overall) | 0.3957 | 0.5200 | 0.4217 | 0.2095 | 0.3403 | 0.2293 |

The panel data regressions estimate the relation between efficiency and market concentration over the period from 2006 to 2020 while controlling for import bank-level and macroeconomic characteristics. The sample includes 225 banks in 18 countries in the MENA region. Banks included in the sample are either conventional banks or Islamic banks. As a measure of bank profitability, we use Return on Equity (ROE) and Net Income Margin (NIM), and for efficiency the measures are DEA efficiency scores (CRS, VRS and SCALE). Market concentration is measured by the HH-Index. Bank-level characteristics and macroeconomic variables are computed as of year *t-1*. All the regressions control for year- and country-fixed effects. *, **, and *** indicate statistical significance at the 10%, 5%, and 1% level, respectively. Bank-level characteristics, institution, ownership, and macroeconomic variables are described in S1 Table.
