# Supplementary material for: Efficiency, market concentration and bank performance during the COVID-19 outbreak: Evidence from the MENA region
Source: PLoS One. 2023 May 10;18(5):e0285403. doi: 10.1371/journal.pone.0285403 (PMC10171612; doi:10.1371/journal.pone.0285403)
Supplement: S1 Fig — (DOCX) [file pone.0285403.s006.docx]

**S1 Fig**

**Fig. 1** below illustrates the relationship between the level of banking market concentration (measured by the HH-index) and efficiency, based on the estimation results obtained from Models 1, 2 and 3 in Table 3. Only statistically significant explanatory variables have been used to determine the efficiency levels and their values. With the exception of the HHI variable, they are equal to sample averages for the period between 2006 and 2020. We run this analysis for the whole sample (Panel A), as well as for the sub-samples of CBs (Panel B) and IBs (Panel C) using the data in Tables 4 and 5. Each panel illustrates a separate chart for the respective efficiency measures (CRS, VRS and SCALE).

**Panel A: All banks (CRS, VRS and SCALE)**

**Panel B: CB sample (CRS, VRS and SCALE)**

**Panel C: IB sample (CRS, VRS and SCALE)**

**Fig. 1 Relationship between bank efficiency and market concentration (HHI)**

**Fig. 2** below illustrates the relationship between the level of banking market concentration (measured by the CR3 ratio) and efficiency, based on the estimation results obtained from Models 4, 5 and 6 in Table 3. We run this analysis for the whole sample (Panel A), as well as for the sub-samples of CBs (Panel B) and IBs (Panel C) using the data in Tables 4 and 5. Each panel illustrates a separate chart for the respective efficiency measures (CRS, VRS and SCALE).

**Panel A: All banks (CRS, VRS and SCALE)**

**Panel B: CBs sample (CRS, VRS and SCALE)**

**Panel B: IBs sample (CRS, VRS and SCALE)**

**Fig. 2 Relationship between bank efficiency and market concentration (CR3)**

The inverted U-shape curve illustrated on Figure S5.1 and S5.2 means that an increase in concentration, in the case of low and high concentrated markets, provides less incentives for the improvement of banks’ efficiency, contrary to the case of moderately concentrated markets. The maximum point of the parabola corresponds to the optimal concentration level and the maximum bank efficiency, which can be derived from the zero value of the first derivative of the profit efficiency with respect to the concentration. The average value of concentration index for the period between 2006 and 2020 is lower than the values for which the efficiency reaches its maximum (for both groups of banks and for the three efficiency models). Therefore, banks in the MENA countries may improve their efficiency by increasing the level of banking market concentration.
